# FGF9 promotes mouse spermatogonial stem cell proliferation mediated by p38 MAPK signalling

**DOI:** 10.1111/cpr.12933

**Published:** 2020-10-26

**Authors:** Fan Yang, Eoin C. Whelan, Xuebing Guan, Bingquan Deng, Shu Wang, Jiachen Sun, Mary R. Avarbock, Xin Wu, Ralph L. Brinster

**Affiliations:** ^1^ State Key Laboratory of Reproductive Medicine Nanjing Medical University Nanjing Jiangsu China; ^2^ Department of Biomedical Sciences School of Veterinary Medicine University of Pennsylvania Philadelphia Pennsylvania USA

**Keywords:** fibroblast growth factor, niche, self‐renewal, spermatogonial stem cell

## Abstract

**Objectives:**

Fibroblast growth factor 9 (FGF9) is expressed by somatic cells in the seminiferous tubules, yet little information exists about its role in regulating spermatogonial stem cells (SSCs).

**Materials and Methods:**

*Fgf9* overexpression lentivirus was injected into mouse testes, and PLZF immunostaining was performed to investigate the effect of FGF9 on spermatogonia in vivo. Effect of FGF9 on SSCs was detected by transplanting cultured germ cells into tubules of testes. RNA‐seq of bulk RNA and single cell was performed to explore FGF9 working mechanisms. SB203580 was used to disrupt p38 MAPK pathway. p38 MAPK protein expression was detected by Western blot and qPCR was performed to determine different gene expression. Small interfering RNA (siRNA) was used to knock down *Etv5* gene expression in germ cells.

**Results:**

Overexpression of *Fgf9* in vivo resulted in arrested spermatogenesis and accumulation of undifferentiated spermatogonia. Exposure of germ cell cultures to FGF9 resulted in larger numbers of SSCs over time. Inhibition of p38 MAPK phosphorylation negated the SSC growth advantage provided by FGF9. *Etv5* and *Bcl6b* gene expressions were enhanced by FGF9 treatment. Gene knockdown of *Etv5* disrupted the growth effect of FGF9 in cultured SSCs along with downstream expression of *Bcl6b*.

**Conclusions:**

Taken together, these data indicate that FGF9 is an important regulator of SSC proliferation, operating through p38 MAPK phosphorylation and upregulating *Etv5* and *Bcl6b* in turn.

## Graphical abstract

Fibroblast growth factor 9 (FGF9) is expressed by somatic cells in the seminiferous tubules, yet little information exists about its role in regulating spermatogonial stem cells (SSCs). Our data indicate that FGF9 is an important regulator of SSC proliferation, operating through p38 MAPK phosphorylation and upregulating *Etv5* and *Bcl6b* in turn.

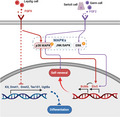

## 1 INTRODUCTION

Spermatogonial stem cells (SSCs) serve as the foundation of mammalian spermatogenesis. SSCs have the capacity to self‐renew or differentiate to form all the cell types in the germ cell lineage.^1^ SSC homeostasis is largely governed by the surrounding niche.^2^ Sertoli, Leydig and myoid cells constitute the majority of the niche and secrete growth factors to regulate SSC homeostasis. Sertoli cells and myoid cells secrete glial‐derived neurotropic factor (GDNF), a potent self‐renewal factor for SSCs.^3^ Leydig cells also produce colony‐stimulating factor 1 (CSF1), which increases stem cell number.^4^ However, there is still much unknown about niche regulating SSC functions.

The fibroblast growth factor (FGF) family plays an important role in the maintenance of SSCs. In humans, dysregulation of signalling pathways involved in SSC regulation by FGF receptor mutations produces a striking paternal age effect through disproportionate self‐renewal of harbouring cells increasing the proportion of mutant spermatozoa.^5^, ^6^, ^7^ FGF2 is required for normal spermatogenesis and is typically included in germ cell culture media as it increases SSC proliferation.^8^, ^9^, ^10^, ^11^ FGF4 enhances regeneration of testis after damage,^12^ and FGF5 also promotes proliferation in culture of spermatogonia expressing GDNF family receptor alpha 1 (GFRα1).^13^ However, much remains unknown about the effect of other FGFs expressed in testes on SSC regulation, such as FGF9. FGF9 expression is observed in the interstitial regions of mice from 14 to 18 days post coitum, and from postnatal days 35 to 65 in Leydig cell cytoplasm, spermatid nucleus and spermatogonium cytoplasm.^14^ Immunohistochemical staining shows FGF9 presence in Leydig cells of 9‐month‐old sheep^15^ and adult humans.^16^ Infertile human patients with Sertoli cell‐only syndrome show decreased FGF9 expression,^16^ suggesting a potential link between aberrant FGF9 expression and normal germ cell maintenance. In culture, FGF9 increases growth of rat^17^ and mouse^18^ germ cells. FGF9 has been suggested to be an inhibitor of meiosis *in vitro*.^19^, ^20^ However, none of these studies have analysed the effect of FGF9 on SSCs via transplantation. Much remains to be understood about the role of FGF9 within the SSC niche, and as FGF9 knockout results in a complete male‐to‐female sex reversion,^21^ its effects on spermatogonia in vivo on the adult testis have not been determined experimentally.

Determining SSC niche growth factors is vital to understand SSC homeostasis and spermatogenesis. In this paper, we determine FGF9 as an SSC growth factor using in vitro culture and transplantation. We show that FGF9 acts as a stem cell renewal factor, inhibits differentiation in vivo when overexpressed and investigate pathways involved in FGF9‐mediated regulation of SSC proliferation.

## 2 MATERIALS AND METHODS

### 2.1 Cell culture

THY‐1^+^ germ cell cultures were established as described previously.^8^ Briefly, male C57 LacZ pups (5‐8 days post‐partum) were sacrificed and testes were digested using Trypsin‐EDTA (Gibco, USA). Cells were magnetically sorted using CD90.2 beads (Miltenyi, USA) and plated onto Sandos inbred mouse (SIM)–derived 6‐thioguanine‐resistant and ouabain‐resistant (STO) feeders (SNL76/7, ATCC). Germ cell cultures were maintained in a humidified atmosphere at 37°C contains 5% CO_2_ with Mouse serum‐free media (mSFM) with 20 ng/mL recombinant human GDNF (R&D, USA), 150 ng/mL recombinant rat GFRα1 (R&D) and 1 ng/mL recombinant human FGF2 (Corning, USA). Other reagents were used as indicated: recombinant human FGF9 (R&D), SB203580, GW788388 and Tofacitinib citrate (all from MedChemExpress, USA). Germ cell counts were determined with a hemocytometer. Feeder cells were identified and discounted from counts based on morphology.^22^ SSC number was calculated using the following formula, adapted from reference ^4^:

$$SSCgrowth=\frac{\mathrm{Cellsharvested}}{\mathrm{Cellsseeded}}\times \frac{\mathrm{Colonies}}{10^{5}cellstransplanted}$$

Here, ‘cells seeded’ indicates the number of cultured THY‐1^+^ germ cells seeded at the beginning of each time period and ‘cells harvested’ is the cell count at the end. ‘Colonies’ indicates the number of distinct colonies counted per testis and is divided by the number of cultured THY‐1^+^ germ cells transplanted per testis.

### 2.2 Transplantation

Transplantation was performed as described previously.^23^ Briefly, cultured THY‐1^+^ germ cells were prepared and injected into the tubules of testis. 2 months later, mice were sacrificed and testes were fixed and stained with X‐gal solution. Tissue processing, sectioning and staining with nuclear fast red was performed by the histology services core at the School of Veterinary Medicine, University of Pennsylvania.

### 2.3 Single‐cell RNA‐seq and bioinformatics

Established THY‐1^+^ germ cells (p12) were plated onto STO feeders 3 days before the start of the experiment and cultured in mSFM with normal growth factors. At hour 0, the cells were switched to mSFM with 20 ng/mL of FGF9 or 0.1% BSA. Cells were cultured for 48 hours, and germ cell clumps were removed by gentle pipetting. Clumps were treated with 0.25% Trypsin for 2 minutes to digest to single cells and strained in a 40 μm filter and blocked in PBS‐S for 10 minutes. Cells were incubated at 4℃ with BioLegend TotalSeq c‐KIT antibody (CITE‐sEquation ^24^) following the manufacturer's protocol. Cell viability following antibody incubation was 89% and 95% for control and FGF9‐treated samples, respectively. Cells were encapsulated and libraries generated using a Chromium Next GEM Single Cell 3’ Kit v3.1 with Feature Barcoding (10X Genomics) per manufacturer's protocol. One replicate of each treatment was encapsulated using cells derived from three individual mice. Libraries were sequenced on a NextSeq 500 sequencer (Illumina) using a 75‐cycle high‐output sequencing kit to a depth of 30k reads per cell. Data were processed using Cell Ranger (10X Genomics) using Mouse reference mm10 (GENCODE vM23/Ensembl 98). Gene counts were analysed with Seurat v3.1^25^ for clustering, integration and differential gene expression and Monocle v3^26^ for pseudotime. Velocyto.R was used to generate RNA trajectories.^27^ For regression of cell cycle genes, gene lists from Macosko *et al* 2015^28^ were used with Seurat's CellCycleScoring function. For the mapping of spliced ratio of transcripts, log‐transformed counts of unspliced were divided by log‐transformed spliced counts for each cell and then transformed back.

### 2.4 Supplementary methods

Additional methods are shown in supplementary material.

## 3 RESULTS

### 3.1 *Fgf9* overexpression increased PLZF‐positive cells in vivo

**FIGURE 1:**
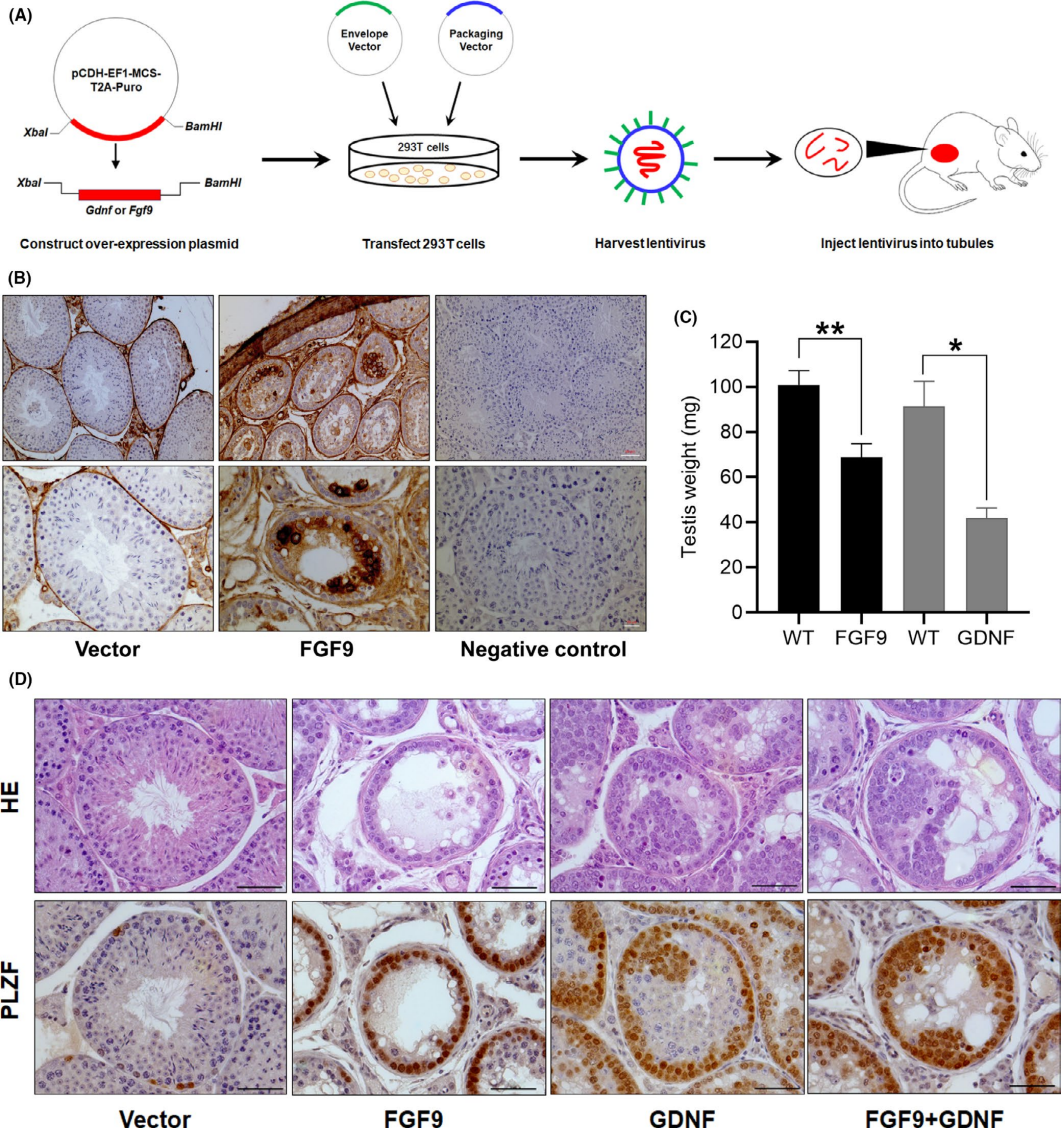
Effect of *Fgf9* and *Gdnf* overexpression plasmids on mouse testes. A, Schematic of experimental design. Two lentiviral plasmids were created containing cDNA of *Gdnf* or *Fgf9* under the control of the ubiquitous promoter EF1. 293T cells were transfected alongside the packaging vector with one of three treatments: *Gdnf* vector, *Fgf9* vector or empty vector. Lentiviral particles were harvested from the 293T cells and injected into the seminiferous tubules of recipient mice. B, FGF9 IHC staining of seminiferous tubule sections from testes infected with overexpression plasmids for 11 wk. Negative control: sections without primary antibody. Scale bar: 50 µm (top) or 20 µm (bottom). C, 11 wk after injection, testes were collected and weighed (FGF9‐overexpression lentivirus injection, n = 5; GDNF overexpression lentivirus injection, n = 3). *P < .05, **P < .01, Student's *t* test. D, Histology and PLZF IHC staining of seminiferous tubule sections from testes infected with overexpression plasmids. Scale bar: 50 µm

**FIGURE 2:**
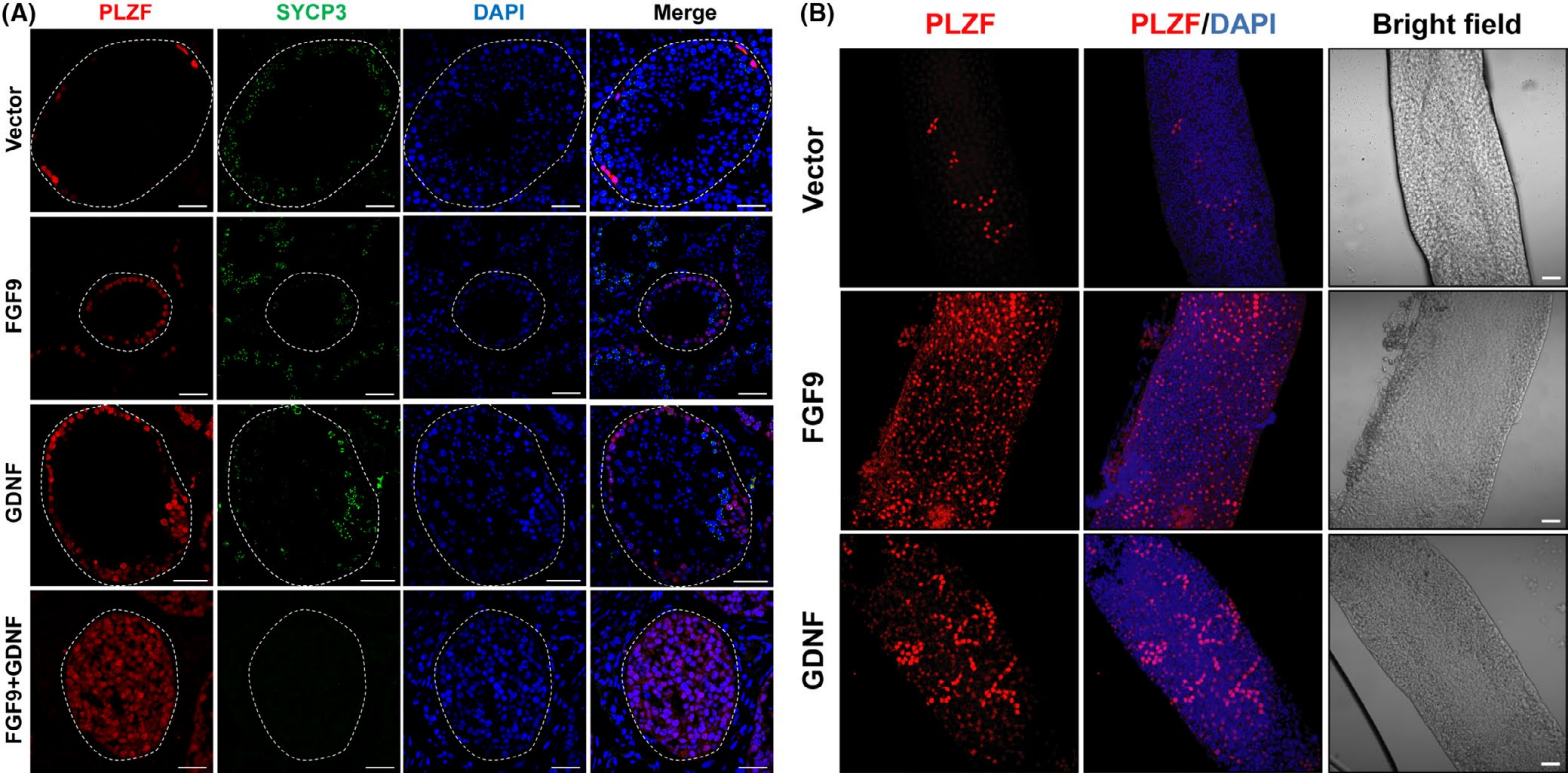
Immunofluorescence of seminiferous tubules exposed to *Fgf9* and *Gdnf* overexpression. A, Cross‐sections of seminiferous tubules stained with antibodies against PLZF (marker for undifferentiated spermatogonia), SYCP3 (essential for meiosis), DAPI (DNA stain) following injection of vector, *Fgf9* and *Gdnf* overexpression plasmids in addition to both overexpression plasmids injected together. Scale bar: 50 µm. B, Whole‐mount staining for PLZF and both PLZF and DAPI of seminiferous tubules injected with vector, *Fgf9* overexpression and *Gdnf* overexpression plasmids. Seminiferous epithelial cycle of all tubules showed here are stages II–VI as determined by transillumination technique. Scale bar: 50 µm

To investigate the role of FGFs in governing SSC self‐renewal, we constructed overexpression lentiviruses containing *Fgf3*, *Fgf5*, *Fgf8* and *Fgf9* and injected into recipient testes (Figure 1A). Only *Fgf9* expression showed dramatic changes in tubule morphology (Figure S1 A). As shown in Figure 1B, FGF9 was mostly expressed in the Leydig cells of control testes injected with empty vector. When the testes were infected with the *Fgf9* overexpression plasmid, somatic cells including Leydig cells and germ cells of later stage all expressed FGF9 signalling. Testes infected with *Gdnf* or *Fgf9* overexpression plasmids for 11 weeks were substantially reduced in size relative to the uninfected testis from the same animal (Figure 1C, Figure S1B and Figure S2). Testes injected with empty vector showed normal histology and PLZF staining of undifferentiated germ cells along the basement membrane (Figure 1D). *Fgf9* overexpression resulted in large numbers of PLZF^+^ cells and few synaptonemal complex protein 3^+^ (SYCP3^+^) cells (Figure 2A). *Gdnf* overexpression produced an increase in PLZF^+^ cells around the basement membrane but also in aggregates within the tubule (Figure 1D). PLZF^–^ differentiating cells were visible within the tubule up to round spermatids, including some SYCP3^+^ cells (Figure 2A). When both *Fgf9* and *Gdnf* were overexpressed, a mixture of both phenotypes was seen: all cells showed strong PLZF staining (Figure 1D), no differentiating or SYCP3^+^ cells were seen and undifferentiated spermatogonia form clumps (Figure 2A). When FGF9 was overexpressed, far more whole‐mount staining of PLZF^+^ cells was visible, whereas GDNF overexpression resulted in even more PLZF^+^ cells with sporadic clumps (Figure 2B and Figure S3). These results suggest that excess FGF9 promotes undifferentiated spermatogonia accumulation and leads to a lack of differentiating cell types in vivo.

### 3.2 Stimulation of SSCs proliferation by FGF9

**FIGURE 3:**
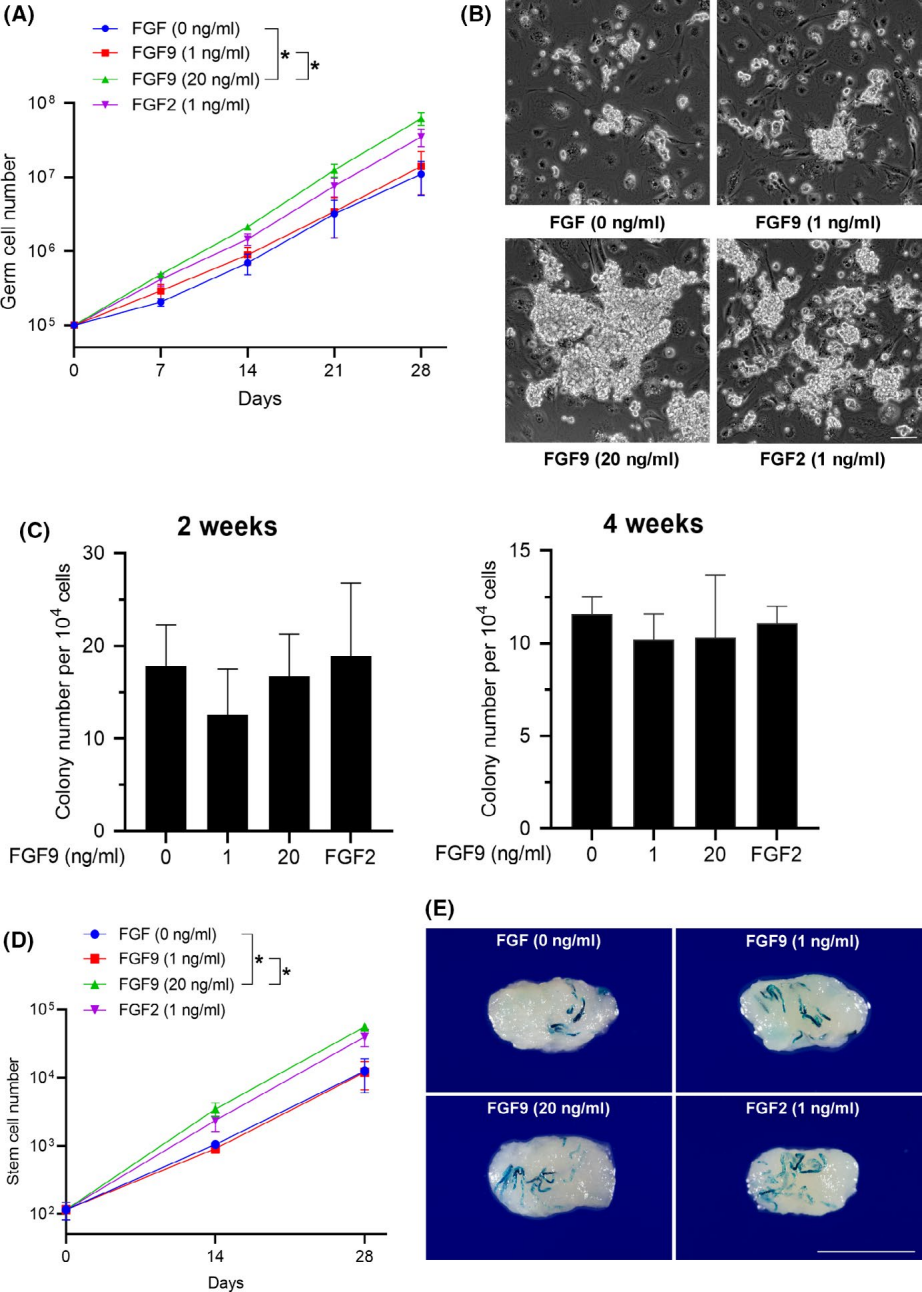
Effect of FGF9 on germ cell proliferation and SSC proliferation. A, Effect of FGF9 and FGF2 treatment on THY‐1^+^ germ cell number in culture. Cells were treated with GDNF, GFRα1 and listed concentration of FGF (n = 3). *P < .05, ANOVA of final cell numbers. B, Representative morphology of cultured THY‐1^+^ germ cell clumps after treatment for 7 days. Scale bar: 100 µm. C, Colony number per 10^4^ cultured THY‐1^+^ germ cells following transplantation (n = 3, 6 testes per replicate). *P < .05, ANOVA. D, SSC number following 2 and 4 wk in culture (n = 3). *P < .05, ANOVA of final cell numbers. E, Representative morphology of receipt testes which were transplanted with germ cells after treatment for 4 weeks. Scale bar: 5 mm. All error bars show SEM

In order to determine the effect of FGF9 on SSCs, we established THY‐1^+^ germ cultures which enriched SSCs (p7‐p12) and plated THY‐1^+^ germ cells on STO feeder plates in mSFM supplemented with GDNF and GFRα1. Cultures were subjected to four treatments: no FGF, 1 ng/mL FGF2, 1 ng/mL FGF9 and 20 ng/mL FGF9. The germ cell cultures showed exponential increases over time (Figure 3A). FGF2‐treatment resulted in more cell numbers over time compared with control although this did not rise to statistical significance. 1 ng/mL FGF9 showed lower levels of cell growth compared with FGF2, largely indistinguishable from controls. However, 20 ng/mL FGF9 significantly increase cell number over time compared with both control and 1 ng/mL FGF9 cultures. For 1 ng/mL FGF2 and 20 ng/mL FGF9, both treatments produced larger‐sized clumps than control (Figure 3B). To assay the number of SSCs, cultured THY‐1^+^ germ cells enriched for SSCs were transplanted into busulfan‐treated mice, where SSCs produce countable colonies of germ cells carrying the *LacZ* marker gene.^29^, ^30^, ^31^ For a given number of germ cells transplanted, we observed no significant differences in colony count between any of the treatments. This indicated that while FGF9 treatment increased the total cell number it did not alter the stem cell concentration (ie, stem cells per 10^5^ cultured THY‐1^+^ germ cells, Figure 3C). Moreover, testes of all groups showed normal spermatogenesis after transplantation (Figure S4). Taking these observations together, SSC number in culture at the three time points was calculated as described in the Methods section. FGF9 20 ng/mL showed significantly more SSCs after 4‐week culture as compared with 1 ng/mL and control (Figure 3D,E). We can conclude that FGF9 exposure leads to a greater cultured THY‐1^+^ germ cell number and SSC number over time.

### 3.3 p38 MAPK signalling is required for FGF9‐activated SSC proliferation

**FIGURE 4:**
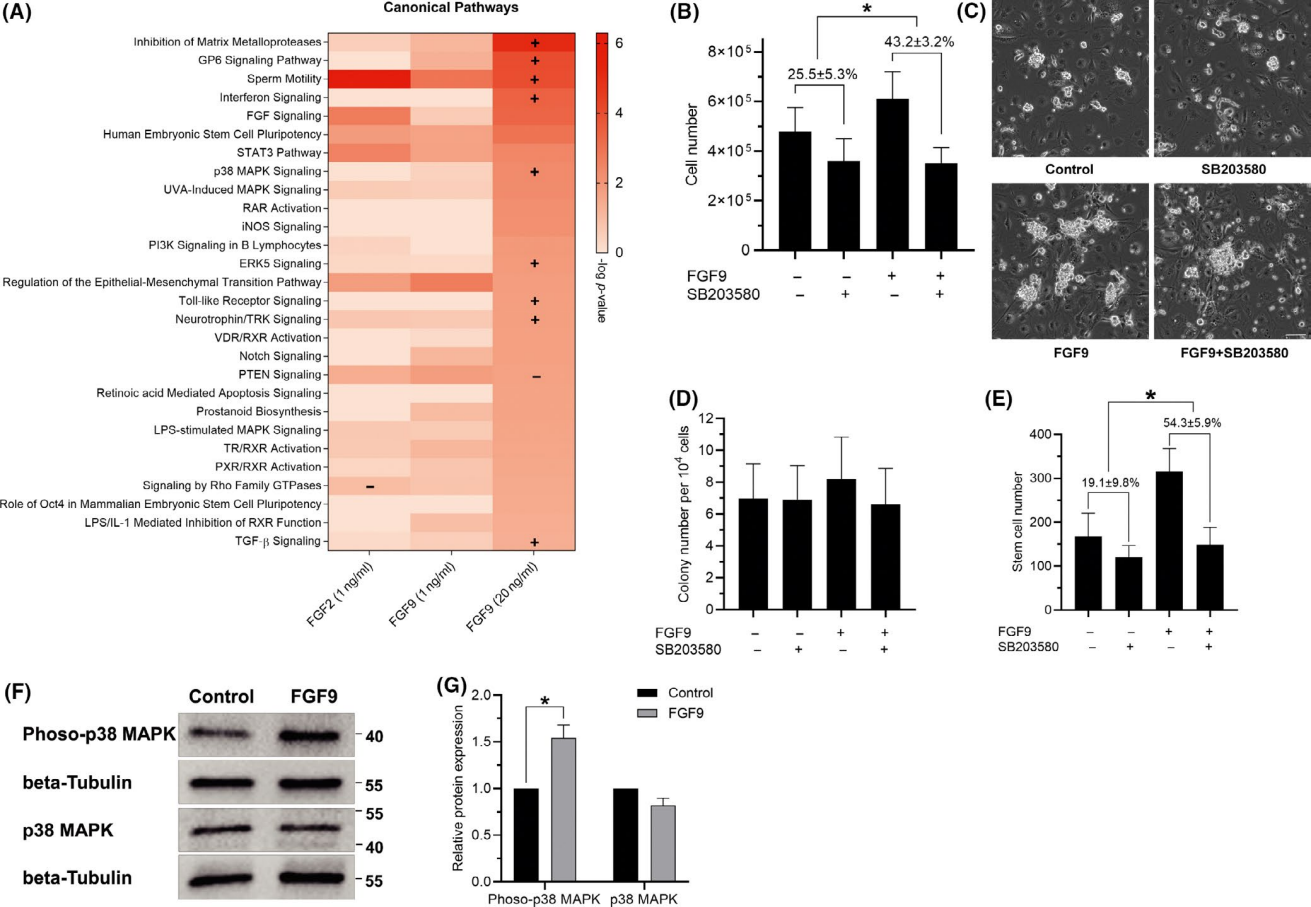
Regulation of p38 MAPK signalling by FGF9 in germ cells. A, Top signalling pathways from Ingenuity Pathway Analysis (Qiagen) of RNA‐seq data. Germ cells were treated with GDNF, GFRα1 and FGFs for 7 d. Then, cells were harvested for RNA‐seq (n = 3). Pathways ordered by 20ng/mL FGF9 *P*‐values. Scale shows ‐log *P*‐value and ‘+’denotes Z‐score of > 1 and ‘‐’ a Z‐score of <−1. B, Effect of SB203580 on germ cell number. Cultured THY‐1^+^ germ cells (ie, heterogeneous cultures of cells including stem cells and spermatogonia) were grown for 7 d in mSFM with GDNF and GFRα1. Cells were additionally treated with 5 μmol/L of SB203580, 20 ng/mL FGF9, neither or both. Percentages show proportional reduction of cell number after 7 d (n = 3). *P < .05, Student's *t* test between proportional germ cell loss. C, Representative morphology of cultured THY‐1^+^ germ cell clumps after treatment for 7 d. Scale bar: 100 µm. D, Colony number per 10^4^ cultured THY‐1^+^ germ cells following transplantation (n = 4, 5 testes per replicate). *P < .05, ANOVA. E, SSC number following 1 wk in culture. Percentages show proportional reduction of cell number after 7 d (n = 4, 5 testes per replicate). *P < .05, Student's *t* test between proportional stem cell losses. F, Protein expressions of phosphorylated‐p38 MAPK and p38 MAPK in germ cells with different treatments. Germ cells were cultured with FGF9 (40 ng/mL) for 2 d and protein was extracted for detection. G, Quantitation of phosphorylated‐p38 MAPK and p38 MAPK protein expressions in germ cells with different treatments (n = 3). *P < .05, Student's *t* test. All error bars show SEM

To explore how FGF9 promotes SSC proliferation, RNA‐seq was performed (Table S3) and validated by qPCR (Figure S5). The pathways that showed the most significance included interferon signalling, p38 MAPK signalling and TGF‐β pathways (Figure 4A). We used pathway inhibitors to verify if disrupting them would negate the proliferative advantage of FGF9 in culture (Figure S6). After seeding cells and exposing cells to control treatment, SB203580 (p38 MAPK pathway inhibitor) reduced the growth of control cells by 25.5 ± 5.3%. FGF9‐treated cultured THY‐1^+^ germ cells grew faster than control, but when FGF9‐treated cells were exposed to SB203580, the growth was reduced by 45.2 ± 3.2%, significantly more than control (Figure 4B,C). FGF9 treatment with and without SB203580 did not substantially alter the colony number per 10^4^ cultured THY‐1^+^ germ cells as compared with unexposed controls (Figure 4D). However, the total number increase of SSCs with FGF9 treatment was largely abolished by SB203580, indicating that disruption of p38 MAPK pathway eliminated the growth advantage conferred by FGF9 (Figure 4E). We found that the phosphorylated p38 MAPK protein significantly increased with FGF9 treatment while the total amount of p38 MAPK did not change (Figure 4F,G). These results show FGF9 promotes SSC proliferation via p38 MAPK pathway.

### 3.4 Regulation of *Etv5* by FGF9

**FIGURE 5:**
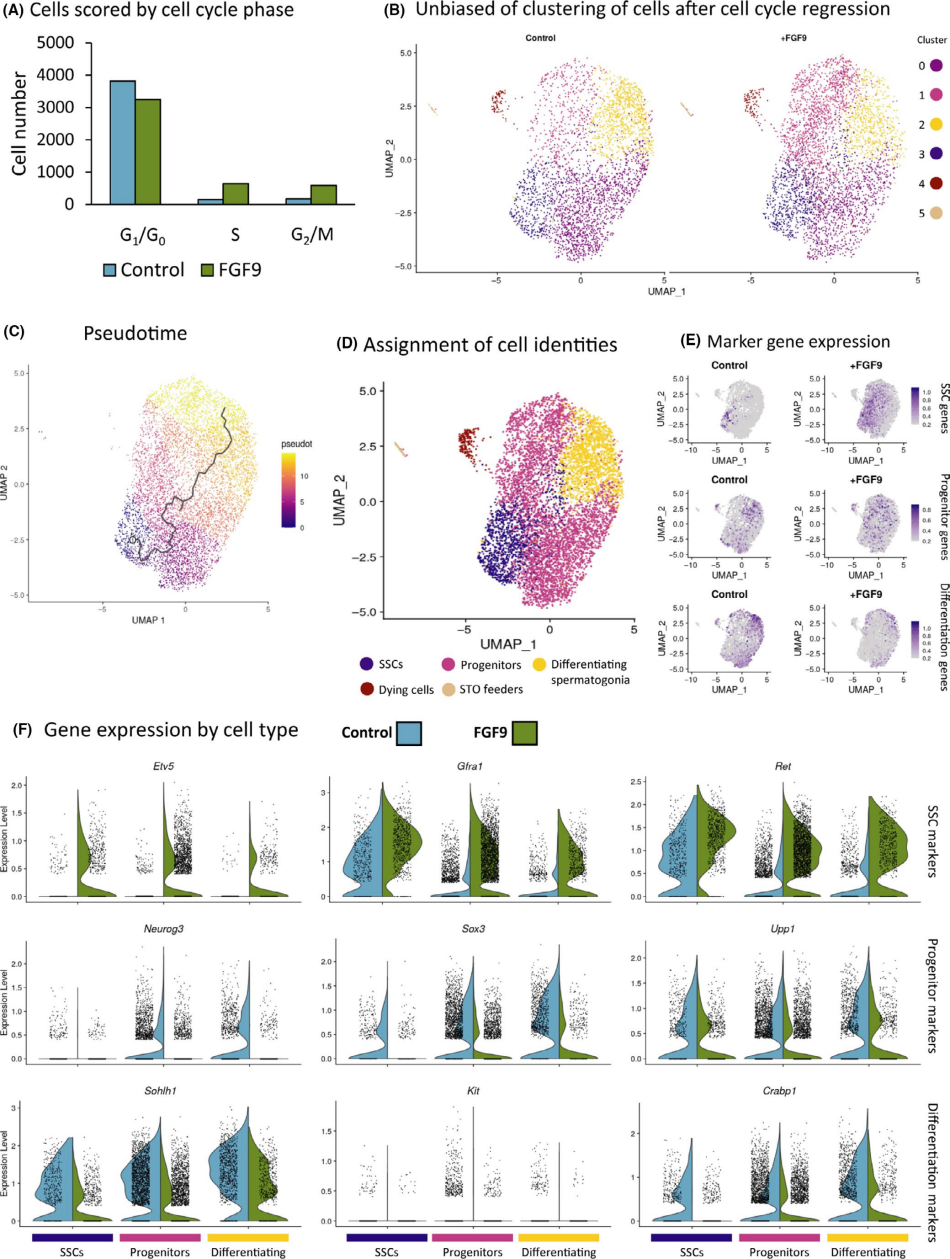
Single‐cell RNA‐sequencing analysis of FGF9 effect on cultured THY‐1^+^ germ cells in vitro. RNA‐sequencing analysis of 4,143 cells cultured for 48 h in mSFM with 20 ng/mL FGF9 integrated with 4482 control cells cultured with no growth factors for the same time. A, Cells clustered and scored for cell cycle expression (see Figure S7C). B, Clustering of cells after regressing out cell cycle genes for S and G_2_M phases. C, Pseudotime trajectory. D, Assignment of unbiased clusters to cell identities indicated by gene expression profile (see Figure S7G for dying cells and STO). Note that ‘SSCs’ are used to designate the cluster containing SSCs, it is likely that not all cells are true SSCs. E, Module scores using sets of marker genes. For SSC: *Gfra1, Ret, Etv5, Id4, Tspan8* and *Esrp1*. For progenitor spermatogonia: *Upp1, Lhx1, Nanos3, Sox3* and *Galnt12*. For differentiating spermatogonia, *Kit, Sohlh1, Crabp1* and *Lmo1*. F, Violin plots of gene expression by cell type and treatment. Three genes were selected as representative markers of gene expression for each of the three cell identities

While conventional bulk RNA‐seq provided pathways perturbed by FGF9, cells in culture represent a heterogeneous population, only some of which are stem cells capable of reconstituting a niche. In order to determine whether FGF9 affects a subset of cells within the culture, established THY‐1^+^ germ cells were treated with FGF9 and single‐cell RNA‐seq was performed. After filtering, 4482 FGF9‐treated cells were sequenced with a median 13,289 unique molecular identifiers (UMIs) and 4,300 genes. The control sample contained 4,143 cells with a median 14,927 UMIs and 4,626 genes (Figure S7B). Cells were clustered and scored for RNA velocity and cell cycle phase (Figure S7A and C). Cells in S or G_2_/M phases clustered together, and the RNA velocity was dominated by cell cycle changes. FGF9‐treated cells showed a higher proportion of cells in S or G_2_/M (Figure 5A) and modestly higher RNA velocity (Figure S7A). Only a small number of contaminating STO feeder cells were detected (Figure S7G). To remove the effect of cell cycle on clustering, cell cycle genes were regressed out and the cells reclustered. Cells showed substantial cluster overlap between treatments (Figure 5B). Unbiased clustering showed four clusters, assigned to three cell types via pseudotime trajectory (Figure 5C) and pattern of gene expression (Figure 5E and Figure S7L). While it is important to note cells in vitro may not match exactly to developmental stages in the testis, clusters were defined as ‘SSCs’, that is, the most undifferentiated cells that displayed stem cell markers such as E26 variant transcription factor 5 (*Etv5*) and *Gfra1*; ‘progenitors’ being intermediate cells showing undifferentiated markers such as neurogenin 3 (*Neurog3*); and ‘differentiated spermatogonia’ as cells showing the earliest markers of differentiating spermatogonia (Figure 5D,E and Figure S7L). In each case, the control cells were used for assessment of markers. FGF9 treatment in SSCs was associated with both higher expression and more cells expressing stem cell marker genes such as *Etv5*, rearranged during transfection (*Ret*) and *Gfra1* and significantly lower expression of *Sox3*, spermatogenesis and oogenesis specific basic helix‐loop‐helix 1 (*Sohlh1*), and ubiquitin C‐terminal hydrolase L1 (*Uchl1*, Figure 5F and Figure S7I). The SSC compartment also contained more cells in S or G_2_M phases after FGF9 treatment: 60 out of 666 (9.0%) FGF9‐treated cells compared with 8 out of 442 control cells (1.8%, Figure S7D‐F). Interestingly, higher expression of SSC marker genes was also observed in progenitor and differentiating germ cells (Figure 5F), suggesting that FGF9 treatment results in higher expression of *Etv5* even in progenitor/differentiating cells. The upregulation of stem cell marker genes like *Etv5* is also associated with both an increase in unspliced transcript counts (Figure S7K) and unspliced to spliced ratio of these genes (Figure S7H), suggesting that the observed increase in gene expression is due to enhanced transcription. *Stra8* is a gene upregulated by retinoic acid‐induced differentiation^32^ but does not show a difference between treatment groups, indicating this pathway has not been activated in differentiating cells in culture. While SSC marker expression was more prevalent across differentiating cell types after treatment, 80% of FGF9‐treated cells nevertheless clustered with progenitor or differentiating control cells. The presence of the receptor KIT was also assayed, and while *Kit* expression and KIT epitope patterning were relatively weak, control cells nevertheless showed higher expression of both RNA and protein than FGF9‐treated cells (Figure S7J).

**FIGURE 6:**
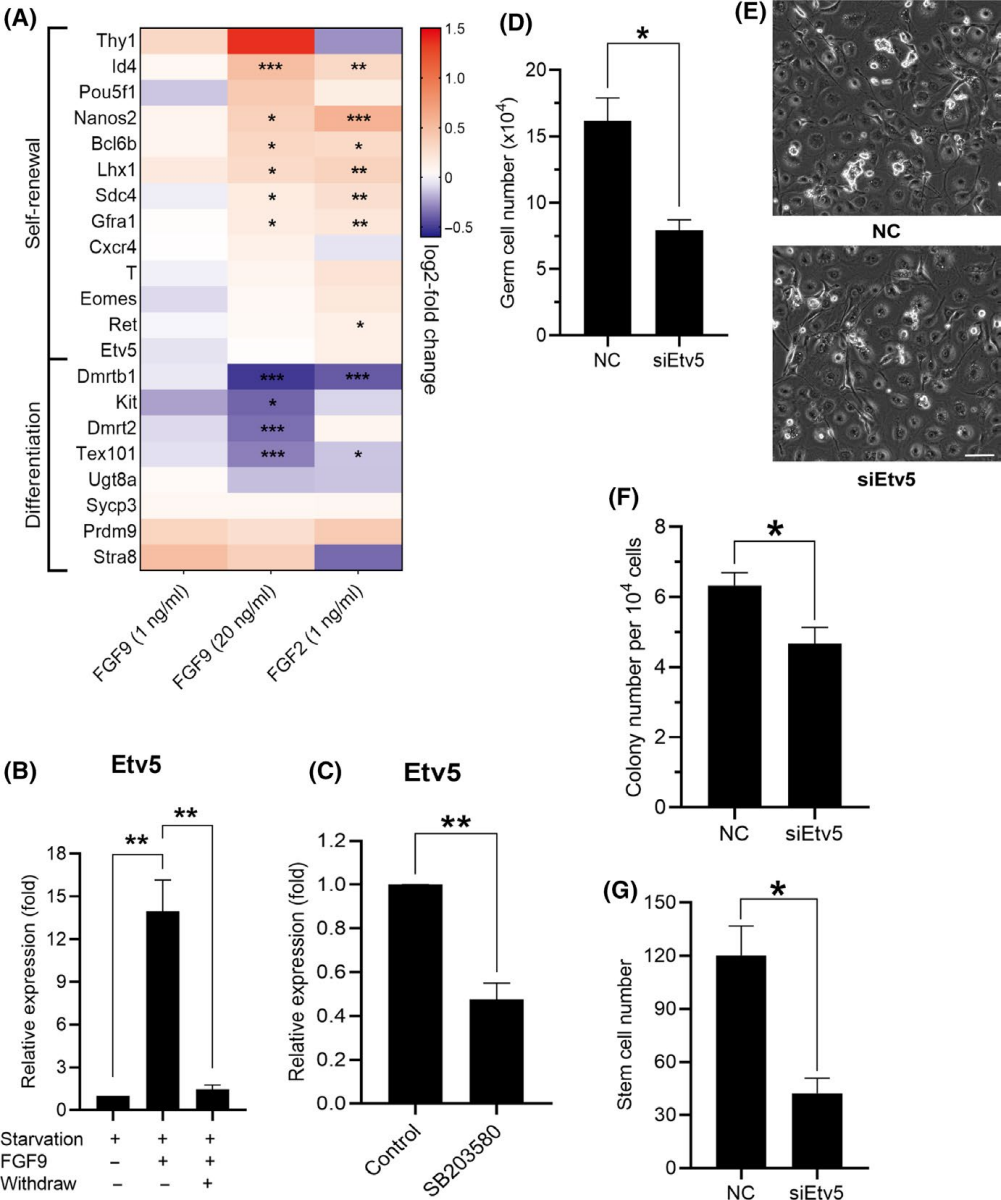
Regulation of *Etv5* by FGF9 in germ cells. A, Heat map of selected self‐renewal and differentiation genes. Scale shows log_2_‐fold change. *P < .05, **P < .01, ***P < .001. B, qPCR expression values of *Etv5* (n = 3). Starvation treatment: cells cultured in mSFM with no growth factors for 2 d. FGF9 treatment: cells treated with mSFM containing 20 ng/mL of FGF9 for 2 d. Withdraw treatment: cells cultured in mSFM with no growth factors for 2 d. **P < .01, ANOVA. C, qPCR analysis of *Etv5* expression. Cells were cultured with FGF9 or FGF9 with SB203580 (30 μmol/L) for 3 d and then harvested for qPCR analysis (n = 3). **P < .01, Student's *t* test. D, Effect of *Etv5* siRNA on germ cell number. After 24 h of transfection of *Etv5* siRNA, cells were cultured with FGF9 for 6 d (n = 3). *P < .05, Student's *t* test. E, Representative morphology of cultured THY‐1^+^ germ cell clumps after treatment for 6 d. Scale bar: 100 µm. F, Colony number per 10^4^ cultured THY‐1^+^ germ cells following transplantation (n = 3, 4 testes per replicate). After 24 h of transfection of *Etv5* siRNA, cells were cultured with FGF9 for 6 d and then transplanted into recipient animals. *P < .05, Student's *t* test. G, SSC number recovered from culture (n = 3, 4 testes per replicate). *P < .05, Student's *t* test. All error bars show SEM

Figure 6A shows the effect of FGFs on the genes known to be involved in self‐renewal or differentiation of spermatogonia (Table S2). Self‐renewal genes show increased expression particularly for 20 ng/mL FGF9 and 1 ng/mL FGF2 treatments. Conversely, differentiation genes show a general trend of downregulation. To validate this effect, cells were cultured for 2 days in mSFM lacking growth factors, treated for 2 days with 20 ng/mL FGF9 and then cultured with mSFM lacking growth factors for 2 days (Figure 6B, ‘starvation’, ‘FGF9’ and ‘withdrawal’, respectively). After treatments, qPCR was performed on the SSC markers *Etv5,* inhibitor of DNA binding 4 (*Id4*), syndecan 4 (*Sdc4*)*, Csf1, Gfra1* and the differentiation marker *Kit*. All SSC markers showed an uptick after FGF9 treatment that returned to baseline after withdrawal; whereas *Kit* showed a downregulation during FGF9 exposure (Figure S8A). Of these, *Etv5* showed the most dramatic response following FGF9 treatment (Figure 6B and Figure S8A). It is notable that *Etv5* did not show significant upregulation in RNA‐seq data (Figure 6A), possibly because the cultures used for RNA‐seq included GDNF and GFRα1 that are known to regulate *Etv5* expression.^9^, ^33^ Results also showed a significant decrease of *Etv5* expression in cells exposed to SB203580 (Figure 6C). To further confirm *Etv5* is required for the effect of FGF9 on cell growth, small interfering RNA (siRNA) was applied. THY‐1^+^ germ cell number in the *Etv5* knockdown group was significantly lower than the control (Figure 6D,E). SSC number per 10^4^ cultured THY‐1^+^ germ cells was reduced by a small, but significant amount (Figure 6F). *Etv5* siRNA treatment reduced total stem cell number significantly by 50% (Figure 6G). These results indicate FGF9 activates *Etv5* through p38 MAPK pathway.

### 3.5 FGF9 activates *Bcl6b* through *Etv5*

**FIGURE 7:**
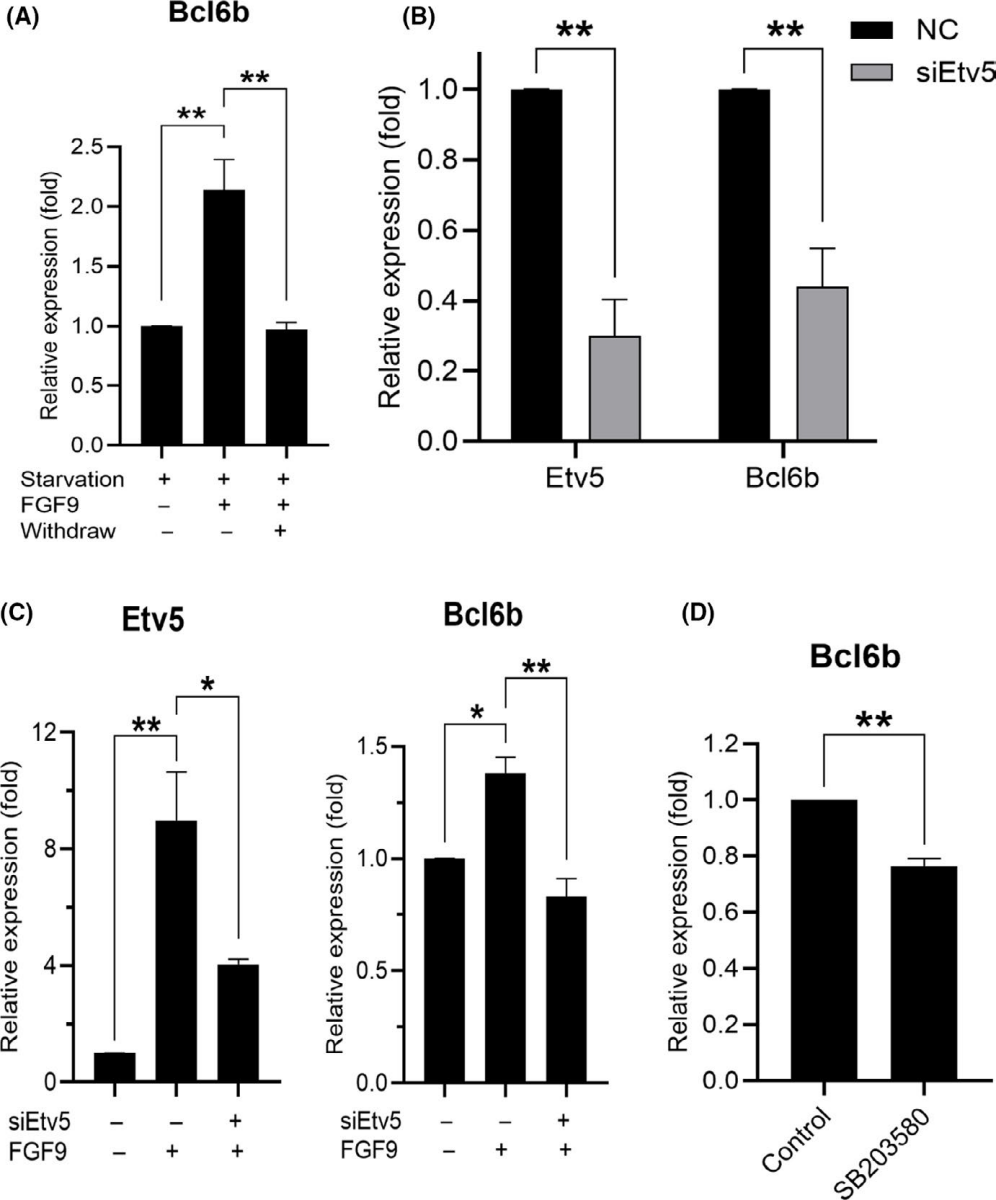
Regulation of *Bcl6b* by *Etv5* after FGF9 activation. A, qPCR expression values of *Bcl6b* (n = 3). Starvation treatment: cells cultured in mSFM with no growth factors for 2 d. FGF9 treatment: cells treated with mSFM containing 20 ng/mL of FGF9 for 2 d. Withdraw treatment: cells cultured in mSFM with no growth factors for 2 d. *P < .05, ANOVA. B, qPCR analysis of *Etv5* and *Bcl6b* expressions in cultured THY‐1^+^ germ cells after 24 h of transfection with *Etv5* siRNA (n = 3). *P < .05, Student's *t* test. C, qPCR analysis of *Etv5* and *Bcl6b* expressions in cultured THY‐1^+^ germ cells. After 24 h of transfection of Etv5 siRNA, cells were starved for 2 d and treated with FGF9 for 2 d (n = 3). *P < .05, **P < .01, ANOVA. D, qPCR analysis of *Bcl6b* expression. Cells were cultured with FGF9 or FGF9 with SB203580 (30 μmol/L) for 3 d and then harvested for qPCR analysis (n = 3). **P < .01, Student's *t* test. All error bars show SEM

We selected LIM homeobox 1 (*Lhx1*), brachyury (*T*) and B‐cell CLL/lymphoma 6 member B (*Bcl6b*) as candidates to investigate the downstream effect of *Etv5* (Figure S8B).^34^, ^35^ Of these, only *Bcl6b* showed a concomitant upregulation with *Etv5* when cultured THY‐1^+^ germ cells were exposed to FGF9 (Figure 7A). There was a significant decrease of *Bcl6b* expression following *Etv5* knockdown (Figure 7B). When treated with FGF9, *Bcl6b* expression was indeed reduced when *Etv5* was knocked down (Figure 7C). In addition, we saw a significant decrease of *Bcl6b* expression in cultured THY‐1^+^ germ cells when treated with SB203580 (Figure 7D). These results demonstrated that p38 MAPK phosphorylation induced by FGF9 regulates *Bcl6b* expression via *Etv5* in germ cells.

## 4 DISCUSSION

The FGF family plays an important role in diverse physiological processes, both in embryonic development and as growth factors throughout life.^36^ Many FGFs are expressed in the testis along with FGF receptors.^37^ FGF signalling is intimately tied to SSC self‐renewal. The FGF receptors FGFR2 and FGFR3 show striking paternal age effects for the specific mutations that cause Apert's syndrome and achondroplasia, respectively, and these mutations show compelling evidence for clonal expansion of SSCs through inappropriate FGF signalling.^38^, ^39^ FGF2 has been known to regulate SSC proliferation, both in culture^8^, ^10^, ^11^ and in vivo.^40^ While FGF2 has been extensively studied, much remains to be known about the role of other FGF proteins expressed within the testis.^36^

The most well‐known growth factor for SSC self‐renewal is GDNF.^41^ In mutant mice containing a *Ret*‐inactivating mutation that disrupts GDNF action, a subset of spermatogonia persist via FGF signalling from Sertoli cells.^18^ This suggests FGFs can stimulate SSC self‐renewal independent of GDNF signalling. PLZF is a marker for undifferentiated spermatogonia,^42^ and in our experiments, WT testes displayed few PLZF^+^ cells, consistent with undifferentiated spermatogonia comprising only 0.3% of germ cells.^43^ Following *Fgf9* stimulation, PLZF^+^ cells were spread around the basement membrane and tubules lacked differentiating cell types, as determined by morphology and SYCP3 expression, a protein involved in the synaptonemal complexes of meiosis.^44^ The observed increased number of PLZF^+^ cells in tubules overexpressing FGF9 along with loss of chains of cells suggests an increase in progenitor spermatogonia and potentially an increase in SSCs.^45^

In order to dissect the effect of FGF9 on SSCs, we turned to our in vitro SSC culture system. SSC number increased with FGF9 dose in a similar manner as FGF2.^10^ Meanwhile, RNA‐seq data indicated that both FGF9 and FGF2 induced similar gene regulations, broadly upregulating SSC self‐renewal genes such as *Nanos2*, *Id4*, *Etv5* and *Bcl6b*.^46^, ^47^, ^48^ This is associated with a downregulation of spermatogonial differentiation genes such as *Dmrt1* and *Kit*,^49^, ^50^ and these changes are consistent with both FGF2 and FGF9 being pro‐stem cell factors. At the single‐cell level, we see that FGF9 enhances expression of *Etv5* along with the other stem cell markers such as *Ret* and *Gfra1* expression in the defined SSCs cluster. We see a pattern of increased stem cell markers across all cell types along with a decrease in differentiation markers. This is evidenced by both more cells within the cluster showing detectable transcripts as well as higher normalized counts, although FGF9‐treated cells continue to cluster with differentiating control cells. This suggests that pro‐stem cell pathways are activated by FGF9 across all cells in culture but only partially prevent differentiation as heterogeneity persists in culture. To support this RNA finding, we detected in cells exposed to FGF9 a reduction of KIT protein on the cell surface in comparison with untreated cells. We also see more cells in S or G2/M phases, suggesting that cell division is promoted and this accounts for the higher cell numbers with FGF9 treatment.

**FIGURE 8:**
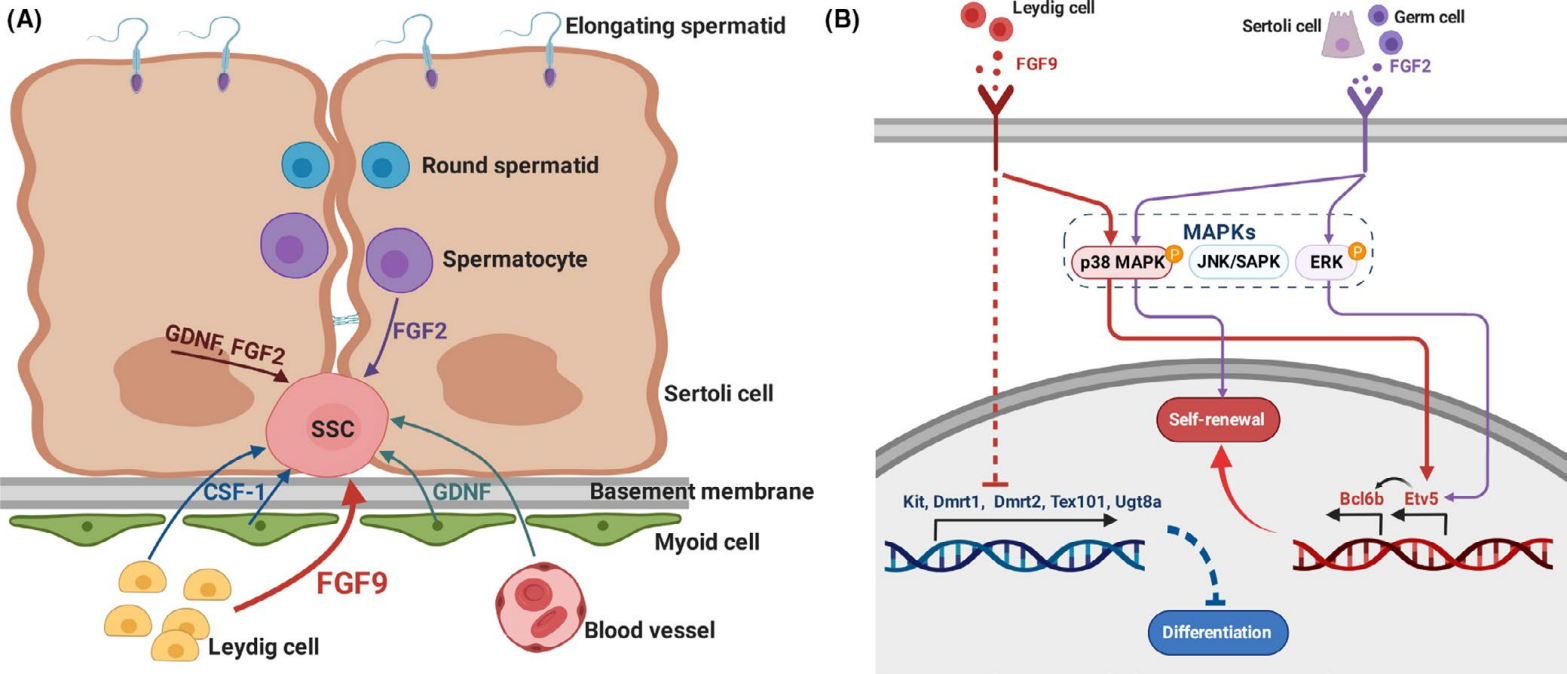
Diagram of proposed action of FGF9. A, Somatic cells provide growth factors to influence cell fate of SSCs. Sertoli cells supply GDNF and FGF2, Leydig cells provide CSF‐1 and FGF9, myoid cells provide GDNF and CSF‐1, blood vessels produce GDNF and FGF2 signalling occurs from differentiating germ cells as well.^2^, ^56^ B, FGF2 induces via FGF receptors (FGFR 1c, 3c > 2c, 1b, 4Δ) both p38 MAPK and ERK signalling pathways that independently lead to proliferation of SSCs via *Etv5* upregulation.^9^, ^36^, ^51^ FGF9 also stimulates FGF receptors (FGFR 3c > 2c> 1c, 3b» 4Δ) with considerable overlap with FGF2,^36^ and we show in this study this leads to p38 MAPK phosphorylation, which activates *Etv5* gene expression as well. After *Etv5* activation, *Bcl6* expression is upregulated, thereby regulating SSC proliferation. Simultaneously, FGF9 exposure inhibits differentiation, ultimately by affecting the expression of pro‐differentiation genes

MAP kinases are divided into three main families, each of which have been reported to be involved in SSC regulation.^9^, ^51^ These are the extracellular signal‐regulated kinases (ERKs), the Jun amino‐terminal kinases/stress‐activated protein kinases (JNKs/SAPKs) and p38 MAP kinase, see Figure 8B.^52^, ^53^ Activation of MAP2K1 from the ERK family in the absence of FGF2 produces an increase in germ cell number,^9^ providing strong evidence that FGF2 operates through the ERK family. However, FGF2 has also been reported to stimulate germ cell p38 MAPK phosphorylation, and p38 MAPK is essential for mouse male germline stem cell self‐renewal.^51^, ^54^ In our results, we found p38 phosphorylation inhibition eliminated the FGF9‐provided growth advantage for both cultured THY‐1^+^ germ cells and SSCs, suggesting an important role of p38 MAPK in the activation of FGFs on SSC self‐renewal. MAP2K1 overexpression in germ cells increases expression of *Etv5* and *Bcl6b*.^9^ *Etv5* is necessary for homeostatic spermatogenesis considering that mice with a knockout of *Etv5* undergo the first wave of spermatogenesis but suffer a steady loss of germ cells over time.^55^ siRNA treatment against *Etv5* reduces SSC self‐renewal in vitro and *Bcl6b* expression,^34^ indicating *Bcl6b* is downstream of *Etv5*. siRNA knockdown of *Bcl6b* reduces SSC number in culture and targeted deletion in mice results in abnormal morphology of seminiferous tubules, reduced testis size and compromised fertility.^47^ Given that both *Etv5* and *Bcl6b* are critical for the proper self‐renewal of murine SSCs, we demonstrated here that FGF9 also upregulated both *Etv5* and *Bcl6b* as a response to p38 MAPK phosphorylation. Taken together, FGF2 and FGF9 share many similarities in their effect on germ cells including gene expression profiles, shared pathway activation, growth advantage to germ cell number in culture and SSC proliferation. Some of this effect is likely to be attributed to the overlap in FGF receptor specificity between FGF2 and FGF9; both share an affinity to FGFR3c and FGFR4Δ and, to a lesser extent, FGFR2c and FGFR1c.^36^ Considerable evidence from animal models and cell culture support FGF2 as a niche factor for SSCs.^56^ Given that FGF9 is expressed in the testis and the similarity in germ cell response to FGF2, it is reasonable to suggest that FGF9 is also an SSC niche factor.

Despite the similarities in mechanistic response to FGF2 and FGF9 stimulation, certain differences between the two factors exist. Firstly, FGF2 is expressed via the Sertoli cells and differentiating germ cells,^56^ while FGF9 is mainly expressed in Leydig cells,^14^, ^15^, ^16^ see Figure 8A. The location of undifferentiated spermatogonial populations along the basement membrane allows them to receive signals such as FGF9 from interstitial cells unlike the differentiating cells surrounded by the Sertoli cells within the adluminal compartment. FGF9’s striking effect on SSC proliferation indicates Leydig cells contribute more to SSC regulation than previously shown. Secondly, FGF2 overexpression via lentiviral vectors does not change the proportion of undifferentiated‐to‐differentiated spermatogonia, despite its potent growth capability in culture,^18^ whereas we show FGF9 overexpression results in a near‐complete block of differentiation coupled with expansion of undifferentiated spermatogonia. This picture is complicated by our observation that FGF9 is less potent at equal concentrations than FGF2 in inducing SSC expansion in culture. These results suggest there are differences in the degree to which FGF2 and FGF9 are capable of inducing SSC proliferation in vivo. The fact that FGF2 knockout does not produce a visible phenotype in the testis^57^ implies that other FGFs can compensate for its absence, and FGF9 may be one of these factors.

Understanding how cytokines produced by the niche regulate SSC self‐renewal is vital to our understanding of SSC homeostasis and spermatogenesis. We show FGF9 is a potent niche factor that promotes SSC proliferation. Determining the intracellular mechanisms by which growth factors achieve their control over SSC fate determination is equally important. Our results indicate FGF9 induces phosphorylation of p38 MAPK and activate its signalling cascade within the SSCs. This results in an upregulation of *Etv5* expression, which in turn increases *Bcl6b* expression, ultimately leading to an increased population of stem cells.

## CONFLICT OF INTEREST

None declared.

## AUTHOR CONTRIBUTION

XW, RB, FY and EW designed the study. FY, XG, BD, SW, JS, EW and MA collected the data. FY and EW analysed the data. RB and XW managed the project. EW, FY, RB and XW prepared the manuscript.

## Supporting information

Table S1

Table S2

Table S3

Appendix S1

## Data Availability

The data are available from the corresponding author upon reasonable request.
