# Supplementary material for: FGF9 promotes mouse spermatogonial stem cell proliferation mediated by p38 MAPK signalling
Source: Cell Prolif. 2020 Oct 26;54(1):e12933. doi: 10.1111/cpr.12933 (PMC7791179; doi:10.1111/cpr.12933)
Supplement: Supplementary file 1 — Table S1 [file CPR-54-e12933-s001.docx]

**Primers for lentiviral plasmid construction**

| **Gene** | **Forward** | **Reverse** |
| --- | --- | --- |
| *Gdnf* | GCTCTAGAGCCACCATGAAGTTATGGGATGTC | CGGGATCCGATACATCCACACCGTTTAG |
| *Fgf9* | ATGGCTCCCTTAGGTGAAG | GCTTTGGCTTAGAATATCC |
| *Fgf3* | ATGGGCCTGATCTGGCTTCTG | ACAGCCAGTCCACCTGTATGC |
| *Fgf5* | CCAAGCTGTGACCGGCGCCTACTCTAGAATGAGCCTGTCCTTGCTCTTCCTCATCTTCTGCAGCCACCTG | CTTCGGGAGTGAGACGCTTCTCCCCGTGAGCCCAAGCGCTGTGGATCAGGTGGCTGCAGAA |
|  | GTCTCACTCCCGAAGGGCAACCCGCGCCTCCTAGGAACCCGGGAGACTCCAGCGGCAGCCG | TGGTGAGGAGGCAGAAGACGAAGAAAACGTCGCGCTACTTCTGCCCCGGCTGCCGCTGGAG |
|  | TCTGCCTCCTCACCAGTCGCAGCTTCTCCGGGCAGCCAAGGAAGCGGCTCGGAACATAGCAGTTTC | CTCTGCAGTACAGGCTGCCGGTCCGGCGCCCCGAAGGGCTCCACTGGAAACTGCTATGTTC |
|  | GCCTGTACTGCAGAGTGGGCATCGGTTTCCATCTGCAGATCTACCCGGATGGCAAAGTCAATGGCTCCCA | TACAATCCCCTGAGACACAGCAAATATTTCCAAAATACTTAACACACTGGCTTCGTGGGAGCCATTGACT |
|  | GTCTCAGGGGATTGTAGGAATACGAGGAGTTTTCAGCAACAAATTTTTAGCGATGTCAAAA | TCTCCCTGAACTTACAGTCATCCGTAAATTTGGCACTTGCATGGAGTTTTCCTTTTTTTGACATCGCTAA |
|  | GTAAGTTCAGGGAGAGATTCCAAGAAAACAGCTATAATACCTATGCGTCCGCGATCCACAGAACTGAAAA | GCCTCTCTTGGCTTTCCCTCTCTTGTTCAGGGCCACGTACCACTCTCGGCCTGTCTTTTCAGTTCTGTGG |
|  | AAAGCCAAGAGAGGCTGCAGCCCACGGGTCAAACCCCAACACGTCTCCACCCACTTCCTACCCAGGTTCA | TTTTTCTTTTCTGGAACAGTGACGGTGAAGGAAAGTTCCGGTTGCTCGGACTGCTTGAACCTGGGTAGGA |
|  | TCCAGAAAAGAAAAAGCCACCGGTGAAACCAAAGGTGCCCCTGTCGCAGCCTCGCAGAAGTCCCAGCCCA | CTTCCTCTGCCCTCAGCGGCCGCGGATCCTCCAAAGCGAAACTTCAGTCTGTACTTCACTGGGCTGGGACTTCT |
| *Fgf8* | CCAAGCTGTGACCGGCGCCTACTCTAGAATGGGCAGCCCCCGCTCCGCGCTGAGCTGCC | AGCGCTCCTTACCTGGGCTTGGAGGCAGAGAACCAGCAAGTGCAACAGCAGGCAGCTCAGCGCGG |
|  | CAGGTAAGGAGCGCTGCGCAGAAGCGGGGGCCGGGCGCGGGGAACCCAGCTGACACTCT | CCCTGCTGCGCTGGCCGAAGGGTCGGTCCTCGTGTCCCTGCCCGAGAGTGTCAGCTGGG |
|  | GCCAGCGCAGCAGGGCTGGAAAGAACTTTACAAATCCAGCCCCAAACTACCCCGAGGAGGGATCT | CTCACATGTCGCTGTGTGACTTTAGGCAGGACACTGTCTCTCTGTTCCTTAGATCCCTCCTCGGG |
|  | ACAGCGACATGTGAGGGAGCAGAGCCTGGTGACGGATCAGCTCAGCCGCCGCCTCATCCGGAC | GGACCTGCACGTGCTTCCCGCTGGTGCGGCTGTAGAGCTGGTAGGTCCGGATGAGGCGG |
|  | AGCACGTGCAGGTCCTGGCCAACAAGCGCATCAACGCCATGGCAGAAGACGGAGACCCCTTCGCG | CCGCGAACTCGGACTCTGCTTCCAAAAGTATCGGTCTCCACAATGAGCTTCGCGAAGGGGTCTCC |
|  | AGTCCGAGTTCGCGGCGCAGAGACAGGTCTCTACATCTGCATGAACAAGAAGGGGAAGCTAATTG | CAGCACGATCTCTGTGAATACGCAGTCCTTGCCTTTGCCGTTGCTCTTGGCAATTAGCTTCCCCT |
|  | ACAGAGATCGTGCTGGAGAACAACTACACGGCGCTGCAGAACGCCAAGTACGAGGGCTGGTACAT | GCGTCTTGGAGCCCTTGCGGGGCCGGCCCTTGCGGGTAAAGGCCATGTACCAGCCCTCG |
|  | AGGGCTCCAAGACGCGCCAGCATCAGCGCGAGGTGCACTTCATGAAGCGCCTGCCGCGG | TTGAGGAACTCGAAGCGCAGGCTCTGCTCGGTGGTGTGGTGGCCCCGCGGCAGGCGCTT |
|  | CTTCGAGTTCCTCAACTACCCGCCCTTCACGCGCAGCCTGCGCGGCAGCCAGAGGACTTGG | CTTCCTCTGCCCTCAGCGGCCGCGGATCCTCGGGGCTCCGGGGCCCAAGTCCTCTGGCT |

**qPCR Primers**

| **Gene** | **Forward** | **Reverse** |
| --- | --- | --- |
| *Rps2* | GAGCCTGGGTCCTCTGAACA | CTGACTCCCGACCTCTGGAAA |
| *Etv5* | GAGCCGCTCTCTCCGCTATT | CGTTCCCCAGCCACCTT |
| *Id4* | TGCAGCACGTTATCGACTACATC | TCAGCAAAGCAGGGTGAGTCT |
| *Gfra1* | CAACTTCATGCATATGGCTCTCA | TCTGCTAAAGCACTGGGCTTCT |
| *Sdc4* | GTCCCCGGAGAGTCGATTC | GCACCAAGGGCTCAATCACTT |
| *Csf1* | ATGAGCAGGAGTATTGCCAAGG | TCCATTCCCAATCATGTGGCTA |
| *Lhx1* | CCCAGCTTTCCCGAATCCT | GCGGGACGTAAATAAATAAAATGG |
| *Brachyury/T* | CTGGGAGCTCAGTTCTTTCG | ACCGTGTGTGTCAGTGGTGT |
| *Bcl6b* | TACTTCAAGGCTTCGCCTCTCT | CTACGTGTTCCATCTGCAAATAGG |
| *Kit* | GATGGCGTTCCTCGCCT | GCCCGAAATCGCAAATCTTT |
