# Supplementary material for: FGF9 promotes mouse spermatogonial stem cell proliferation mediated by p38 MAPK signalling
Source: Cell Prolif. 2020 Oct 26;54(1):e12933. doi: 10.1111/cpr.12933 (PMC7791179; doi:10.1111/cpr.12933)
Supplement: Supplementary file 2 — Table S2 [file CPR-54-e12933-s002.docx]

**Raw data of Fig 6A**

|  | **1 ng/ml FGF9 vs 0 ng/ml FGF** | | | **20 ng/ml FGF9 vs 0 ng/ml FGF** | | | **1 ng/ml FGF2 vs 0 ng/ml FGF** | | |
| --- | --- | --- | --- | --- | --- | --- | --- | --- | --- |
| **Symbol** | **log2 Fold Change** | **lfcSE** | **pvalue** | **log2 Fold Change** | **lfcSE** | **pvalue** | **log2 Fold Change** | **lfcSE** | **pvalue** |
| Thy1 | 0.311 | 0.763 | 0.683 | 1.357 | 0.752 | 0.071 | -0.265 | 0.760 | 0.728 |
| Id4 | 0.066 | 0.113 | 0.555 | 0.498 | 0.109 | 0.000 | 0.302 | 0.110 | 0.006 |
| Pou5f1 | -0.137 | 0.272 | 0.614 | 0.415 | 0.268 | 0.122 | 0.142 | 0.270 | 0.597 |
| Nanos2 | 0.094 | 0.153 | 0.541 | 0.366 | 0.149 | 0.014 | 0.582 | 0.149 | 0.000 |
| Bcl6b | 0.094 | 0.151 | 0.533 | 0.323 | 0.151 | 0.032 | 0.299 | 0.151 | 0.047 |
| Lhx1 | 0.173 | 0.128 | 0.175 | 0.292 | 0.127 | 0.022 | 0.352 | 0.127 | 0.006 |
| Sdc4 | -0.043 | 0.081 | 0.600 | 0.160 | 0.081 | 0.050 | 0.257 | 0.081 | 0.002 |
| Gfra1 | 0.017 | 0.063 | 0.789 | 0.158 | 0.062 | 0.011 | 0.204 | 0.062 | 0.001 |
| Cxcr4 | 0.002 | 0.125 | 0.987 | 0.123 | 0.123 | 0.318 | -0.068 | 0.124 | 0.583 |
| T | -0.036 | 0.129 | 0.778 | 0.089 | 0.127 | 0.482 | 0.226 | 0.127 | 0.075 |
| Eomes | -0.091 | 0.124 | 0.464 | 0.057 | 0.123 | 0.643 | 0.189 | 0.123 | 0.123 |
| Ret | -0.025 | 0.061 | 0.688 | 0.051 | 0.061 | 0.399 | 0.133 | 0.061 | 0.028 |
| Etv5 | -0.068 | 0.071 | 0.335 | 0.016 | 0.071 | 0.817 | 0.133 | 0.071 | 0.060 |
| Dmrtb1 | -0.058 | 0.116 | 0.617 | -0.512 | 0.115 | 0.000 | -0.411 | 0.115 | 0.000 |
| Kit | -0.224 | 0.157 | 0.154 | -0.380 | 0.156 | 0.015 | -0.102 | 0.156 | 0.514 |
| Dmrt2 | -0.092 | 0.101 | 0.365 | -0.351 | 0.100 | 0.000 | 0.084 | 0.098 | 0.390 |
| Tex101 | -0.076 | 0.071 | 0.283 | -0.310 | 0.070 | 0.000 | -0.140 | 0.070 | 0.046 |
| Ugt8a | 0.036 | 0.094 | 0.705 | -0.154 | 0.093 | 0.096 | -0.143 | 0.093 | 0.123 |
| Sycp3 | 0.065 | 0.072 | 0.361 | 0.068 | 0.070 | 0.333 | 0.075 | 0.070 | 0.286 |
| Prdm9 | 0.334 | 0.327 | 0.306 | 0.258 | 0.324 | 0.427 | 0.412 | 0.325 | 0.205 |
| Stra8 | 0.498 | 0.289 | 0.084 | 0.373 | 0.283 | 0.187 | -0.367 | 0.294 | 0.212 |
