# Supplementary material for: FGF9 promotes mouse spermatogonial stem cell proliferation mediated by p38 MAPK signalling
Source: Cell Prolif. 2020 Oct 26;54(1):e12933. doi: 10.1111/cpr.12933 (PMC7791179; doi:10.1111/cpr.12933)
Supplement: Supplementary file 4 — Appendix S1 [file CPR-54-e12933-s004.docx]

**Supplementary Material**

**FGF9 Promotes Mouse Spermatogonial Stem Cell Proliferation Mediated by p38 MAPK Signaling**

Fan Yang,^1,2,*^ Eoin C. Whelan,^2,*^ Xuebing Guan,^1^ Bingquan Deng,^1^ Shu Wang,^1^ Jiacheng Sun,^1^ Mary R. Avarbock,^2^ Xin Wu,^1,†^ Ralph L. Brinster^2^

**Methods**

**Bulk RNA-seq and bioinformatics**

C57LacZ THY-1^+^ germ cell cultures (between passage 9–12) were maintained in mSFM with 20 ng/ml GDNF and 150 ng/ml GFRα1 supplemented with one of four treatments: control (0.1% BSA), FGF2 1 ng/ml, FGF9 1 ng/ml, FGF9 20 ng/ml. After 7-day treatment, cells were gently blown off from the feeder, a method that results in 96% germ cell purity^1^. RNA was extracted using miRNAeasy Mini Kit (QIAGEN, Germany). mRNA libraries were created using TruSeq Stranded mRNA kit (Illumina, USA). Finished libraries were sequenced on NextSeq500 (Illumina) using a 75-cycle high-output sequencing kit, to a mean depth of 28 million reads per sample. Sequences were aligned to mouse transcriptome (Ensembl mouse GRCm38)^2^ and genomic mapping was performed using GRCm38.p6 assembly. Differential gene expression lists were created via DESeq2^3^. Ingenuity Pathway Analysis (QIAGEN, https://www.qiagenbioinformatics.com/products/ingenuitypathway-analysis) was performed on differential gene-expression lists using a cutoff minimum fold-change of ±1.5 and maximum *P*-value of 0.05.

**qPCR**

Cultured THY-1^+^ germ cells were gently blown off from the feeder. RNA was extracted with miRNAeasy Kit (QIAGEN). cDNA was created with High Capacity cDNA Reverse Transcription Kit (Applied Biosystems, USA). qPCR was performed using Fast SYBR Green (Applied Biosystems) and gene primers shown in Table S1. *Rps2* was used as an endogenous control.

**RNA interference**

*Etv5* knockdown in THY-1^+^ germ cells was performed as described previously^1^ with minor modifications. TriFECTa RNAi Kit for *Etv5* was purchased from IDT (USA, cat. #308468921). Sequences were 5'-GGCAUGGAAUUUAAGCUCAUAGAAC-3' and 3'-CUCCGUACCUUAAAUUCGAGUAUCUUG-5'. A negative control DsiRNA (IDT) was used as a control. Germ cells were trypsinized and seeded on STO feeders. Cells were maintained in antibiotics-free mSFM containing 20 ng/ml GDNF, 150 ng/ml GFRα1 and 1 ng/ml FGF2 during the transfection process. Lipofectamine RNAiMAX (Invitrogen, USA) and 40 nM siRNA were added to the mSFM during plating and cells were incubated for 24 hours. After that, media was changed to normal mSFM containing growth factors.

**Histology and immunohistochemistry**

Testes were fixed in Hartman’s Fixative (Sigma) for 24 hours before being embedded in paraffin and sectioned 5 μm thick. Section slides were de-paraffinized and stained with hematoxylin-eosin (Sigma). For immunohistochemistry (IHC), de-paraffinized sections were incubated overnight at 4°C with primary antibody against FGF9 (Affinity, cat. #DF9532) or promyelocytic leukaemia zinc finger protein (PLZF) (R&D, cat. #AF2944) in a dilution of 1:100. After being washed with PBS, slides were incubated with Peroxidase-Conjugated Goat Anti-rabbit IgG (H+L) (Zsbio, China, cat. #ZB-2301) or Peroxidase-Conjugated Rabbit Anti-Goat IgG (H+L) (Yeasen, China, cat. # 33701ES60) for 2 hours at room temperature. A DAB kit (Zsbio) was used to perform staining.

**Immunofluorescence**

Frozen testis sections were prepared and washed three times in PBS. Sodium citrate antigen retrieval was performed followed by three more washes in PBS. Sections were blocked with 3% BSA for 2 hours at room temperature. Slides were incubated with primary antibodies against PLZF (R&D, cat. #AF2944) and SYCP3 (Abcam, cat. #ab97672) in a dilution of 1:200 at 4°C overnight. After being washed with PBS, slides were incubated with TRITC AffiniPure Bovine Anti-Goat IgG(H+L) (Jackson ImmunoResearch, USA, cat. #805-025-180) and Cy2-AffiniPure Donkey Anti-Mouse IgG (Jackson ImmunoResearch, cat. #715-225-150) for 2 hours at room temperature. Images were collected by LSM 700 (ZEISS, Germany).

**Western blot assay**

Western blot analysis was performed as described previously^4^. Briefly, THY-1^+^ germ cell clumps were gently blow off and collected. Protein was extracted and separated by SDS-PAGE. Then samples were transferred onto polyvinylidene fluoride membranes. After being blocked by 5% non-fat dry milk (Labscientific, USA), primary and secondary antibodies were incubated with the membrane. Protein detection was performed using ECL Western Blotting Detection System (Bio-rad, USA) and pictures of bands were generated through Image Lab 3.0 software. Quantification of bands was performed through Image J 1.47v software. Primary antibodies: phospho-p38 MAPK (Cell Signaling Technology, USA, cat. #4511T), beta tubulin (Proteintech, USA, cat. #10094-1-AP) and p38 MAPK (Proteintech, cat. #14064-1-AP). Secondary antibody: goat anti-rabbit IgG (H+L) horseradish peroxidase conjugate (Proteintech, cat. #SA00001-2).

**Whole mount staining**

Testes were digested with 1 mg/ml collagenase (YIFEIXUE, China) and 7 mg/ml DNaseI (Bomei, China) to make separated tubules. Tubules were fixed in 4% paraformaldehyde and blocked in 2% BSA containing 0.1% TritonX-100 for 2 hours, then tubule slides were incubated with PLZF Antibody (R&D, cat. #AF2944) at 4°C overnight. After incubation of Rhodamine (TRITC) AffiniPure Donkey Anti-Goat IgG (H+L) (Jackson ImmunoResearch, cat. #705025147) at room temperature for 2 hours, slides were stained with DAPI (Sigma) for 15 minutes. Images were collected by LSM 700 (ZEISS). Stages of seminiferous epithelial cycle were identified using the transillumination technique^5,6^. Based on the light absorption pattern, three groups of tubules were divided: stages II–VI (the strong spot), stages VII–VIII (the dark zone) and stages IX–XI (the pale zone)^6,7^.

**Mouse testis lentiviral injections**

Transfections were performed as described previously^8,9^. To create plasmids for the overexpression of *Fgf3*, *Fgf9* and *Gdnf*, each of the respective cDNAs extracted from C57BL6 mouse testis were cloned into pCDH-EF1-MCS-T2A-Puro vectors (a gift from Dr. Dahua Chen, Chinese Academy of Science, China). Sequences of *Fgf5* and *Fgf8* were generated using gene synthesis service (TSINGKE, China) and then cloned into pCDH-EF1-MCS-T2A-Puro vectors. Gene primers were shown in Table S1. These overexpression plasmids were packaged into lentiviral particles by transfecting 293T cells with pmd-REV, pmd-1G and pmd-LG packaging plasmids (gifts from Dr. Dahua Chen). Lentivirus particles were concentrated at 50000 g at 4℃ for 2.5 hours and resuspended into 100 μl HBSS. 5-week-old C57BL/6 mice were injected with 10 μl lentiviral suspension. 11 weeks later, mice were sacrificed and testes were sampled for analysis.

**Statistical analysis**

All data are expressed as mean ± standard error of the mean (SEM). Student’s *t*-tests and one-way analysis of variance (ANOVA) tests with Tukey *post-hoc* analyses were performed to evaluate statistically significant differences between groups. In all cases *P* < 0.05 was considered statistically significant.

**Animal experiments**

All experiments involving live animals were approved by the Institutional Animal Care and Use Committees (IACUC) of University of Pennsylvania or Nanjing Medical University. All experiments were performed in accordance with the institutional guidelines of University of Pennsylvania and Nanjing Medical University as appropriate.

**Supplementary Figures and Figure legends**

**
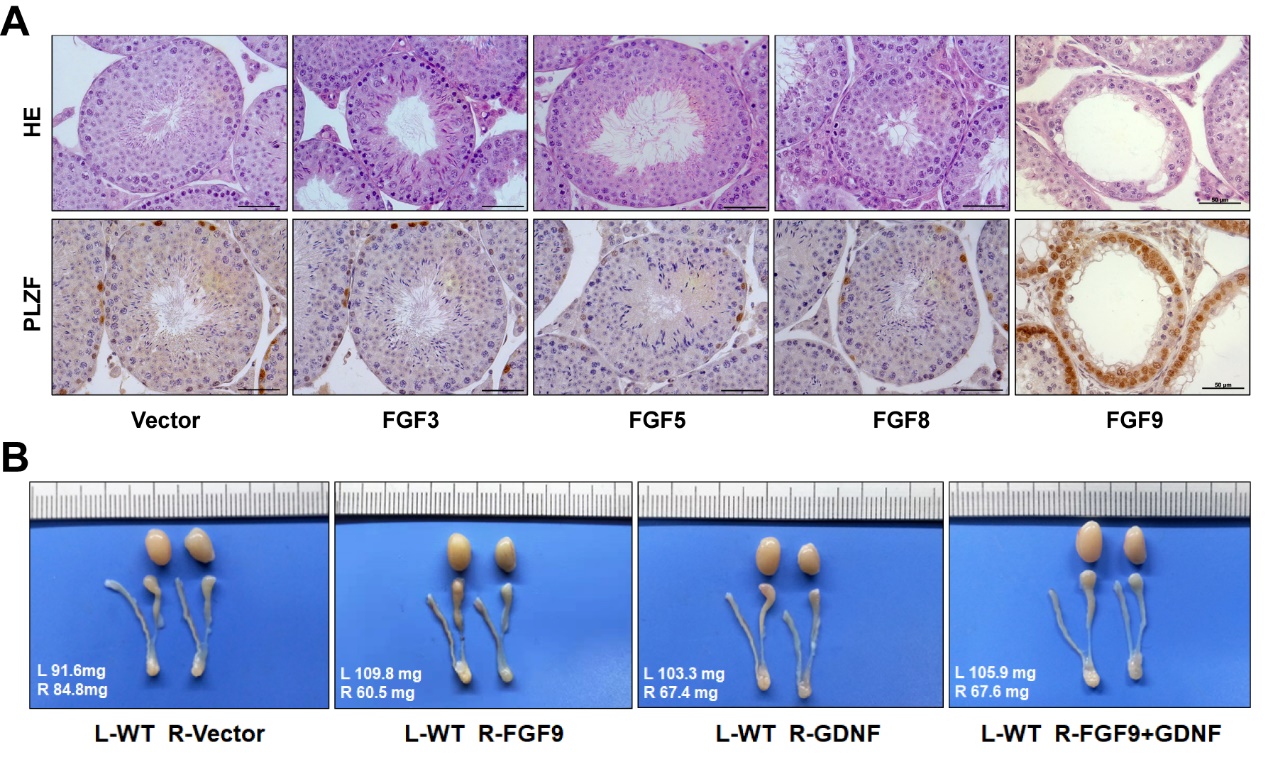
**

**Figure S1. Effect of *Fgfs* overexpression plasmids on mouse testes.**

Testes were injected with overexpression plasmids. **(A)** 6 weeks after injection, testes were fixed and sectioned for histology and PLZF IHC staining. Scale bar: 50 µm. **(B)** Testis morphology following 11 weeks of lentiviral transfection. Testes infected with either *Gdnf* or *Fgf9* overexpression plasmids were compared to the uninfected testis (WT) from the same animal.


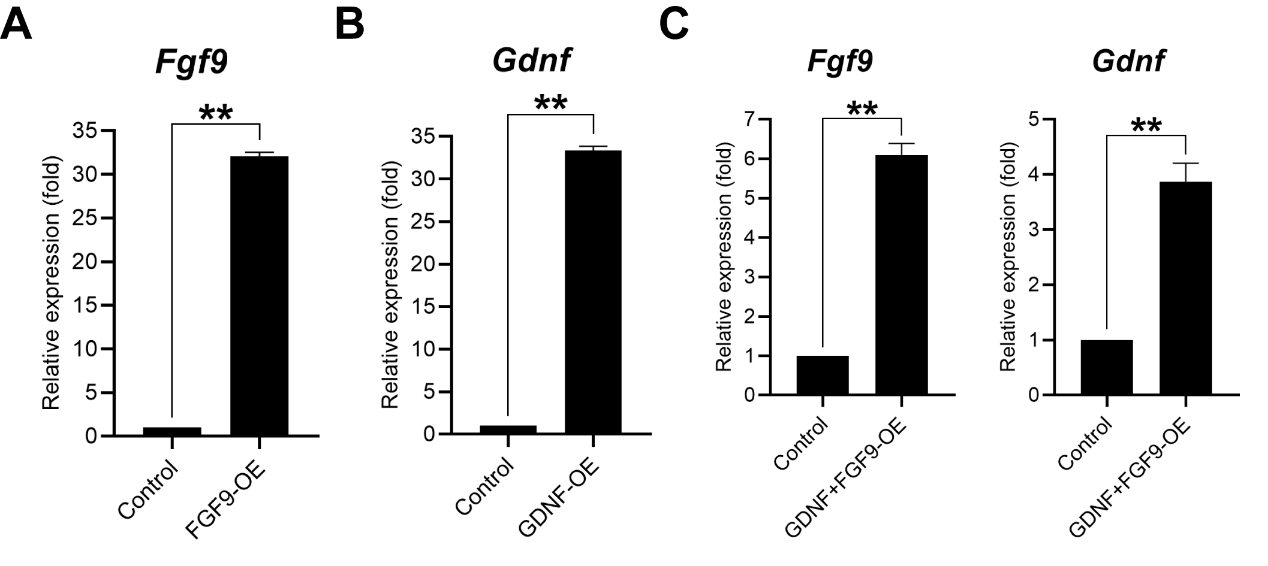


**Figure S2. Expressions of *Fgf9* and *Gdnf* following 11 weeks of lentiviral treatment.**

After 11 weeks after lentiviral injection, testes were collected and total RNA extracted. **(A)** qPCR of *Fgf9* gene expression in mouse testes injected with *Fgf9* overexpression plasmids (n=3). ***P* < 0.01, Student’s *t*-test. **(B)** qPCR of *Gdnf* gene expression in mouse testes injected with *Gdnf* overexpression plasmids (n=3). ***P* < 0.01, Student’s *t*-test. **(C)** qPCR of *Fgf9* and *Gdnf* gene expression in mouse testes transfected with both *Fgf9* and *Gdnf* overexpression plasmids (n=3). ***P* < 0.01, Student’s *t*-test. All error bars show SEM.


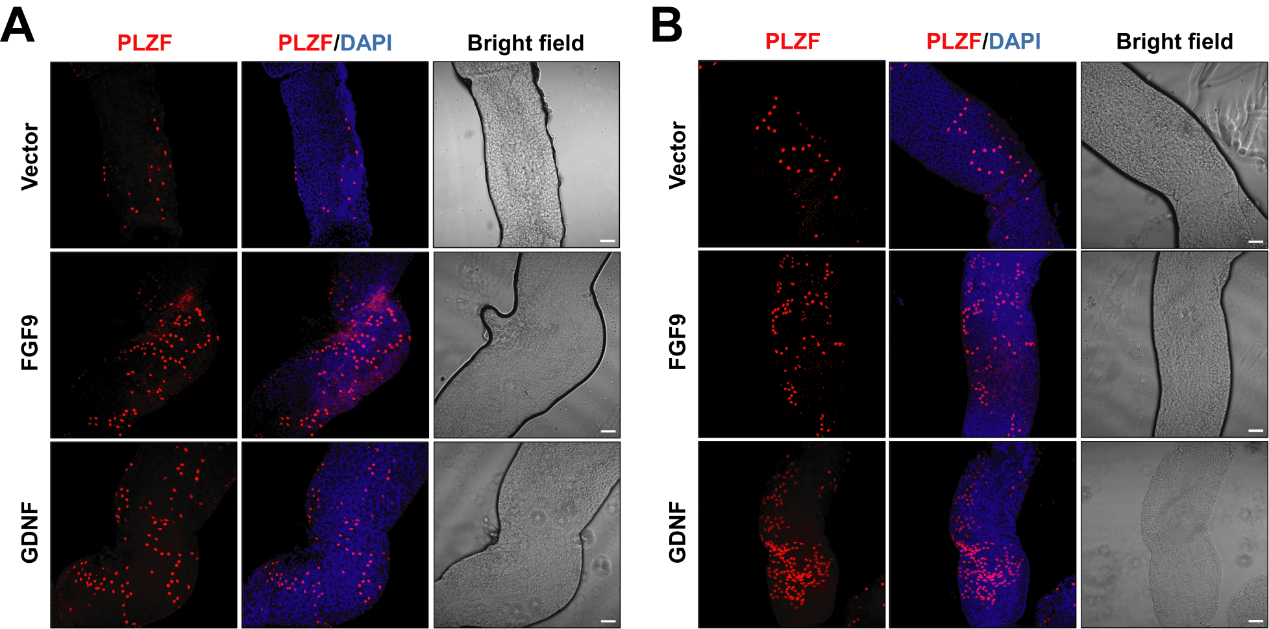


**Figure S3. Immunofluorescence of seminiferous tubules exposed to *Fgf9* and *Gdnf* overexpression.**

PLZF and DAPI whole mount staining of seminiferous tubules injected with vector, *Fgf9*-overexpression, and *Gdnf*-overexpression plasmids. Seminiferous epithelial cycle of all tubules showed here are **(A)** stages VII–VIII and **(B)** stages IX–XI as determined by transillumination technique. Scale bar: 50 µm.


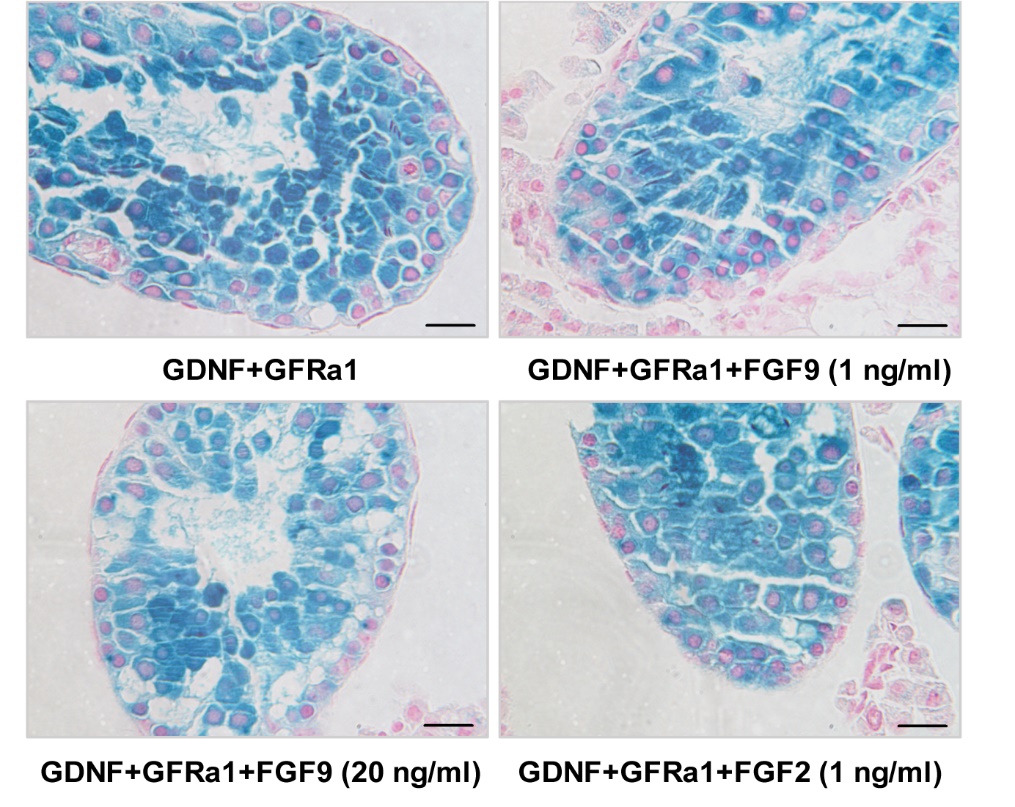


**Figure S4. Histology of seminiferous tubule sections.**

Testes were transplanted with THY-1^+^ germ cells cultured for 4 weeks while supplemented with growth factors as indicated. Testes were removed 8 weeks after transplantation, stained with X-gal solution and sectioned for histology. Scale bar: 20 µm.


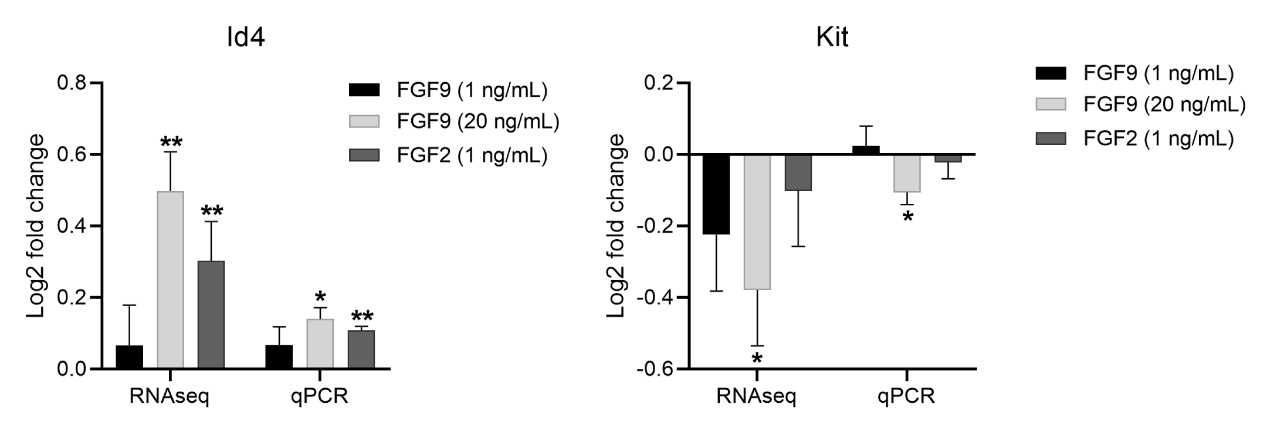


**Figure S5. Validation of RNA-seq data.**

THY-1^+^ germ cells were cultured with GDNF, GFRα1 and FGFs for 7 days. RNA was extracted for RNA-seq and qPCR analyses (n=3). **P* < 0.05, ***P* < 0.01, ANOVA. Error bars show SEM.


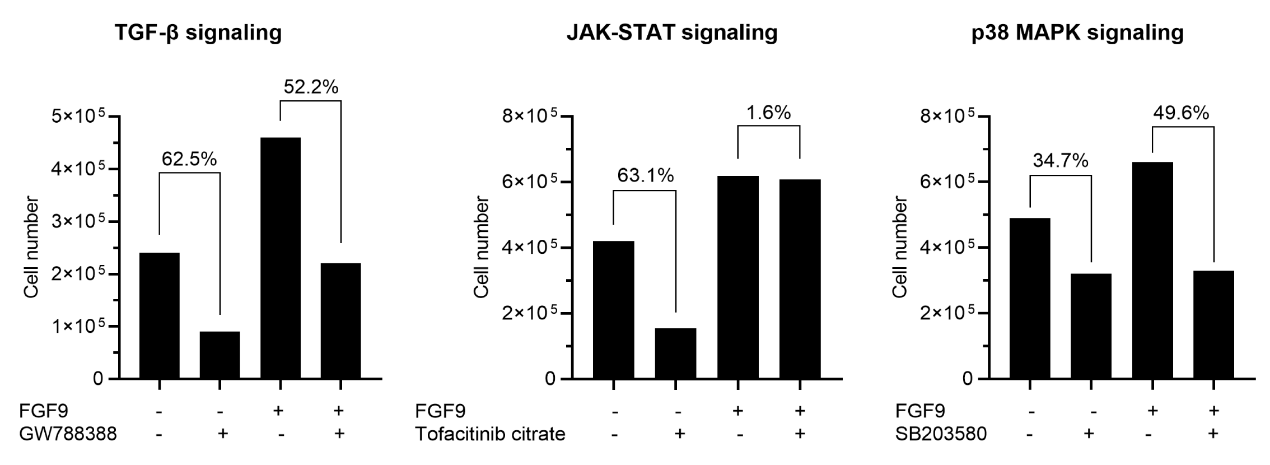


**Figure S6. Effect of pathway inhibitors on cultured THY-1^+^ germ cell number.**

THY-1^+^ germ cells were cultured with 20 ng/ul GDNF, 150 ng/ul GFRα1 plus 20 ng/ul FGF9 and/or one of the following inhibitors as indicated: GW78838 (20 μM, inhibitor for TGF-β), Tofacitinib citrate (10 μM, inhibitor for JAK-STAT signaling) and SB203580 (5 μM, inhibitor for p38 MAPK signaling) for 7 days. Cells were then harvested and counted. Shown is the percentage reduction of cell number with addition of inhibitor (n=1).


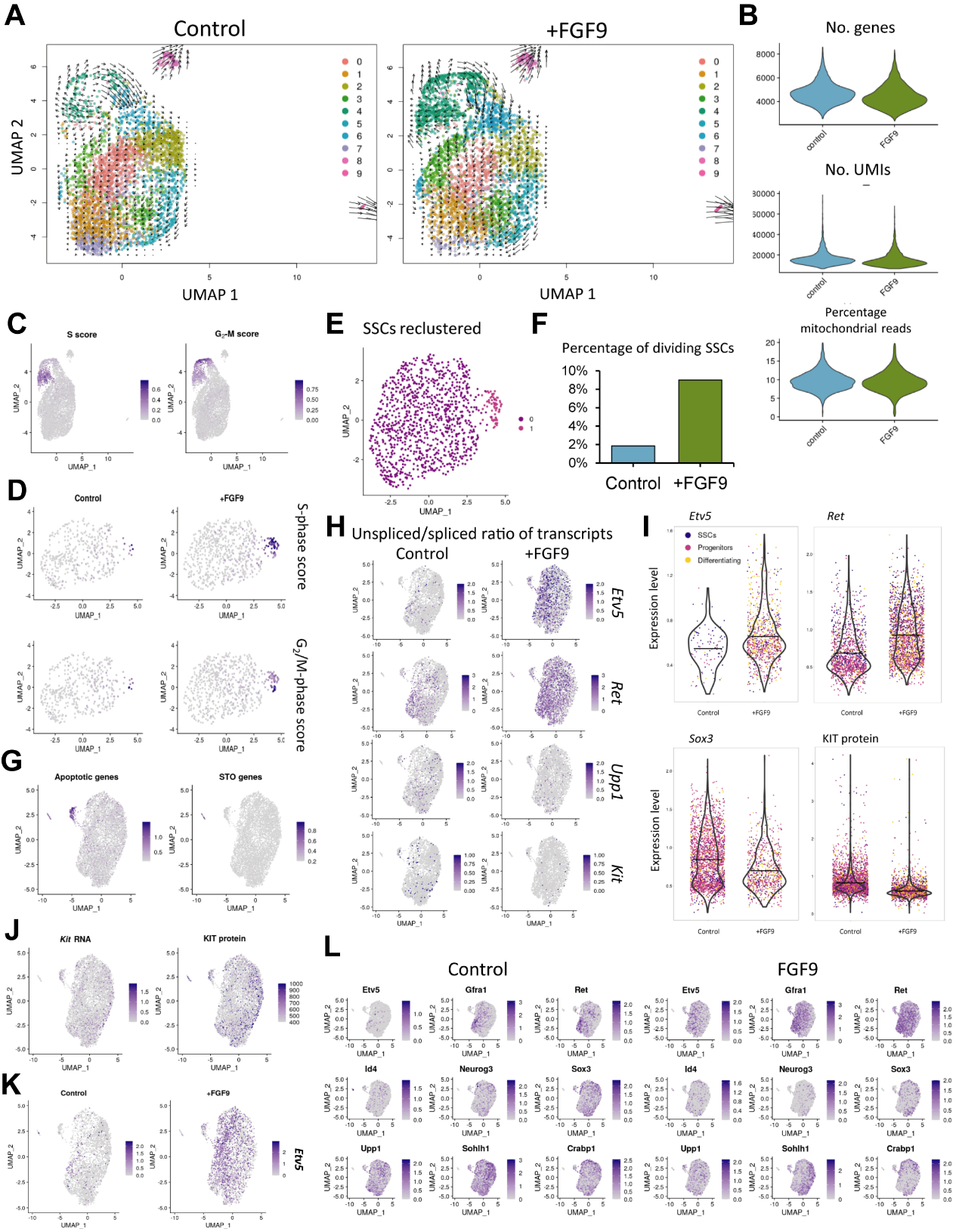


**Figure S7.** **Additional single-cell RNA seq analyses of FGF9 effect on cultured THY-1^+^ germ cells *in vitro.***

**(A)** Unbiased clustering of 8,625 integrated cultured cells treated with FGF9 or vehicle control. RNA trajectory (Velocyto) is shown overlaying clusters (Seurat). **(B)** Gene number, UMI number and percentage of mitochondrial reads across the samples. **(C)** Cell cycle phase scoring via Seurat’s CellCycleScoring function showing cells enriched for cohorts of genes representative of S or G_2_/M phases. **(D)** Cell cycle phase scoring of SSC cluster from Figure 5D. **(E)** Re-clustered SSCs form two clusters, corresponding to G_1_/G_0_ and the other S/G_2_/M. **(F)** The S/G_2_/M-phase cluster comprises 60 out of 666 (9.0%) FGF9-treated cells and 8 out of 442 control cells (1.8%). **(G)** Apoptotic/dying cluster designated with the following apoptotic marker genes *Bax, Ccng1, Trp53inp1, Bbc3, Apaf1, Dpak1.* STO feeder cluster marked with the following genes: *Sox7, Acta2, Thy1.* **(H)** Unspliced/spliced ratio of transcripts shown for two stem cell marker genes and two differentiating genes for each treatment. **(I)** Gene expression of selected genes and KIT protein separated by treatment. Median values are shown as horizontal lines and cells are colored by cell identity. **(J)** Cells expressing *Kit* RNA and KIT surface protein indicated by antibody binding. **(K)** Unspliced transcripts mapped onto UMAP projection for *Etv5.* **(L)** Select germ cell markers by treatment.


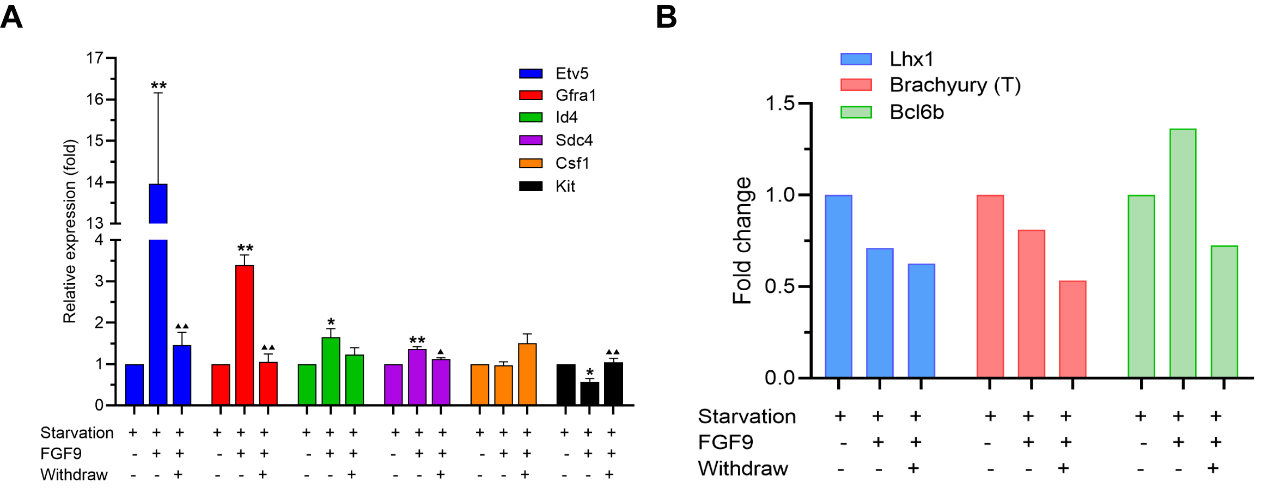


**Figure S8. Effect of FGF9 on gene expressions.**

**(A)** Cultured THY-1^+^ germ cells were starved for 2 days, exposed to FGF9 (20 ng/ml) for 2 days and withdraw FGF9 for 2 days. RNA was extracted for qPCR analysis (n=3). Compared to Starvation group, **P* < 0.05, ***P* < 0.01; compared to FGF9 group, ^▲^*P* < 0.05, ^▲▲^*P* < 0.01, ANOVA. Error bars show SEM. **(B)** Downstream genes of *Etv5* were detected by qPCR (n=1).

**Reference**

1. Oatley JM, Avarbock MR, Telaranta AI, Fearon DT, Brinster RL. Identifying genes important for spermatogonial stem cell self-renewal and survival. *Proceedings of the National Academy of Sciences of the United States of America.* 2006;103(25):9524-9529.

2. Patro R, Duggal G, Love MI, Irizarry RA, Kingsford C. Salmon provides fast and bias-aware quantification of transcript expression. *Nature methods.* 2017;14(4):417-419.

3. Love MI, Huber W, Anders S. Moderated estimation of fold change and dispersion for RNA-seq data with DESeq2. *Genome biology.* 2014;15(12):550.

4. Yang F, Shi L, Liang T, et al. Anti-tumor effect of evodiamine by inducing Akt-mediated apoptosis in hepatocellular carcinoma. *Biochemical and biophysical research communications.* 2017;485(1):54-61.

5. Parvinen M, Vanha-Perttula T. Identification and enzyme quantitation of the stages of the seminiferous epithelial wave in the rat. *Anat Rec.* 1972;174(4):435-449.

6. Grasso M, Fuso A, Dovere L, et al. Distribution of GFRA1-expressing spermatogonia in adult mouse testis. *Reproduction.* 2012;143(3):325-332.

7. Morena AR, Boitani C, de Grossi S, Stefanini M, Conti M. Stage and cell-specific expression of the adenosine 3',5' monophosphate-phosphodiesterase genes in the rat seminiferous epithelium. *Endocrinology.* 1995;136(2):687-695.

8. Wei X, Jia Y, Xue Y, et al. GDNF-expressing STO feeder layer supports the long-term propagation of undifferentiated mouse spermatogonia with stem cell properties. *Scientific reports.* 2016;6:36779.

9. Wang M, Guo Y, Zhou T, et al. The Glial Cell-Derived Neurotrophic Factor (GDNF)-responsive Phosphoprotein Landscape Identifies Raptor Phosphorylation Required for Spermatogonial Progenitor Cell Proliferation. *Molecular & cellular proteomics : MCP.* 2017;16(6):982-997.
